# Addressing vaccine hesitancy: A systematic review comparing the efficacy of motivational versus educational interventions on vaccination uptake

**DOI:** 10.1093/tbm/ibae069

**Published:** 2025-03-26

**Authors:** Sara Labbé, Simon L Bacon, Nana Wu, Paula A B Ribeiro, Vincent Gosselin Boucher, Jovana Stojanovic, Brigitte Voisard, Frédérique Deslauriers, Noémie Tremblay, Lydia Hébert-Auger, Kim L Lavoie

**Affiliations:** Department of Psychology, University of Quebec at Montreal (UQAM), CP 8888, Succursale Centre-Ville, Montreal, Quebec H3C 3P8, Canada; Montreal Behavioural Medicine Centre, Centre Intégré Universitaire de santé et services sociaux du Nord-de-l’Ile-de-Montréal (CIUSSS-NIM), Montreal H4J 1C5, Canada; Montreal Behavioural Medicine Centre, Centre Intégré Universitaire de santé et services sociaux du Nord-de-l’Ile-de-Montréal (CIUSSS-NIM), Montreal H4J 1C5, Canada; Department of Health, Kinesiology & Applied Physiology, Concordia University, Montreal H3G 1M8, Canada; Montreal Behavioural Medicine Centre, Centre Intégré Universitaire de santé et services sociaux du Nord-de-l’Ile-de-Montréal (CIUSSS-NIM), Montreal H4J 1C5, Canada; Department of Health, Kinesiology & Applied Physiology, Concordia University, Montreal H3G 1M8, Canada; Montreal Behavioural Medicine Centre, Centre Intégré Universitaire de santé et services sociaux du Nord-de-l’Ile-de-Montréal (CIUSSS-NIM), Montreal H4J 1C5, Canada; Department of Psychology, University of Quebec at Montreal (UQAM), CP 8888, Succursale Centre-Ville, Montreal, Quebec H3C 3P8, Canada; Montreal Behavioural Medicine Centre, Centre Intégré Universitaire de santé et services sociaux du Nord-de-l’Ile-de-Montréal (CIUSSS-NIM), Montreal H4J 1C5, Canada; Montreal Behavioural Medicine Centre, Centre Intégré Universitaire de santé et services sociaux du Nord-de-l’Ile-de-Montréal (CIUSSS-NIM), Montreal H4J 1C5, Canada; Department of Psychology, University of Quebec at Montreal (UQAM), CP 8888, Succursale Centre-Ville, Montreal, Quebec H3C 3P8, Canada; Montreal Behavioural Medicine Centre, Centre Intégré Universitaire de santé et services sociaux du Nord-de-l’Ile-de-Montréal (CIUSSS-NIM), Montreal H4J 1C5, Canada; Department of Psychology, University of Quebec at Montreal (UQAM), CP 8888, Succursale Centre-Ville, Montreal, Quebec H3C 3P8, Canada; Montreal Behavioural Medicine Centre, Centre Intégré Universitaire de santé et services sociaux du Nord-de-l’Ile-de-Montréal (CIUSSS-NIM), Montreal H4J 1C5, Canada; Department of Psychology, University of Quebec at Montreal (UQAM), CP 8888, Succursale Centre-Ville, Montreal, Quebec H3C 3P8, Canada; Montreal Behavioural Medicine Centre, Centre Intégré Universitaire de santé et services sociaux du Nord-de-l’Ile-de-Montréal (CIUSSS-NIM), Montreal H4J 1C5, Canada; Department of Psychology, University of Quebec at Montreal (UQAM), CP 8888, Succursale Centre-Ville, Montreal, Quebec H3C 3P8, Canada; Department of Psychology, University of Quebec at Montreal (UQAM), CP 8888, Succursale Centre-Ville, Montreal, Quebec H3C 3P8, Canada; Montreal Behavioural Medicine Centre, Centre Intégré Universitaire de santé et services sociaux du Nord-de-l’Ile-de-Montréal (CIUSSS-NIM), Montreal H4J 1C5, Canada

**Keywords:** vaccine hesitancy, behavior change techniques, motivational interviewing, health behavior, systematic review, meta-analysis

## Abstract

Traditional approaches to increase vaccination rely upon educating patients about vaccines. However, research shows that “knowing” vaccines are important is often insufficient: patients need to *believe* that getting vaccinated is important. Evidence-based motivational approaches, such as motivational interviewing/communication (MI/MC), have become increasingly popular for promoting good health behaviors, including vaccination. The objective of this review was to compare the efficacy of educational and MI/MC interventions on vaccination rates relative to each other and to usual/standard care. Pubmed, PsycINFO, and Cochrane trials databases were searched to identify articles that assessed vaccination rates post-patient education or MI/MC vaccine counseling in the context of adult or child vaccination (PROSPERO: CRD42019140255). Following the screening, 118 studies were included (108 educational and 10 MI/MC). The pooled effect sizes for vaccination rates corresponded to 52% for educational interventions (95% CI: 0.48–0.56) and 45% for MI/MC interventions (95% CI: 0.29–0.62) (*P* = .417). Fifty-nine randomized controlled studies (55 educational and 4 MI/MC) showed that, compared with usual/standard of care, exposure to education and MI/MC was associated with a 10% (RR =1.10; 95% CI =1.03–1.16, *P* = .002) and 7% (RR =1.07; 95% CI =0.78–1.45, *P* = .691) increased likelihood of getting vaccinated, respectively. Results suggest comparable efficacy of educational and MI/MC interventions on vaccination uptake and a small superiority of educational interventions compared with usual/standard of care. The overall poor quality of the studies, including lack of fidelity assessments of MI/MC studies, contributes to low confidence in the results and highlights the need for better quality intervention trials examining the efficacy of MI/MC for vaccine uptake.

## Teaser

Both education and motivational counseling are equally effective for improving vaccine uptake, but the strength of the effect for education was weak. The strength of the effect for motivational approaches was uncertain due to the paucity of research, suggesting the need for more research in this area.

**Implications**

Research: Development and delivery of interventions to increase vaccination should be reported with sufficient information to allow replication and determine whether failure/success is related to the hypothesis or the intervention.

Policy: The modest efficacy of education-only interventions should be known among policymakers in order to adapt and optimize strategies aimed at increasing vaccination uptake within the population.

Practice: Healthcare practitioners should explore the potential efficacy of motivational approaches, in addition to education, to more effectively address vaccination hesitancy.

## Background

Vaccination is among the safest and most effective interventions for the prevention of multiple infectious diseases [1, 2]. Over the last two centuries, vaccination has allowed the eradication of smallpox, a life-threatening disease, and prevented the death of millions of people worldwide every year [2]. The recent COVID-19 pandemic illustrates the undeniable impact of vaccination on public health. More than 13 billion doses of COVID-19 vaccines have been administered worldwide, which contributed to an important decrease in SARS-Cov2 virus-related hospitalizations, complications, and deaths with few adverse side effects [3, 4]. Despite their safety, effectiveness, and availability, millions of adults and children remain unvaccinated each year despite clear medical recommendations to receive vaccines. For example, in Canada, childhood immunization coverage is estimated to be 84%, which is far below the target of 95% [5].

In the last decade, a phenomenon called *vaccine hesitancy* has attracted the attention of researchers and public health authorities. In 2019, just prior to the COVID-19 pandemic, the World Health Organization (WHO) listed vaccine hesitancy as one of the 10 most significant threats to global health. Vaccine hesitancy has been defined as: “a delay in acceptance or refusal of vaccination despite availability of vaccination services. [It] is complex and context-specific, varying across time, place and vaccines… [and]is influenced by factors such as complacency, confidence and convenience” [6]. Specifically, the Strategic Advisory Group of Experts (SAGE) on Immunization has identified three broad categories of psychological and context-related determinants that contribute to vaccine hesitancy: (i) complacency regarding personal and collective risks of vaccine-preventable disease; (ii) lack of confidence in the vaccine and the system(s) that administer them; and (iii) the inconveniences associated with getting vaccinated, including issues related to access and/or cost. Successful vaccine strategies should, therefore, include evidence-based interventions that target these factors. However, current approaches to promote vaccination generally focus on providing information (about the benefits of vaccines) and reminders (to both patients and providers) rather than addressing specific psychological or instrumental factors underlying vaccine hesitancy [7].

In 2015, Canadian researchers conducted a review of reviews published between 2008 and 2014 that aimed at identifying the various types of interventions deployed to address vaccine hesitancy in the context of child vaccination and their efficacy. Authors found that education (primarily) is the most common intervention *used* to increase vaccination coverage [7]. Results failed to identify any superiority of a particular intervention type on vaccination. Another systematic review performed by the SAGE group in 2015 also identified educational interventions as the most common type of intervention used to increase vaccination uptake [8]. Although providing health information to educate patients is recognized as the responsibility of healthcare providers (HCPs), this is often done in a unidirectional way that reflects an “expert-patient relationship” instead of a collaborative partnership using an open, nonjudgmental approach that may be better adapted to manage the beliefs and concerns typically held by those with vaccine hesitancy [9]. Moreover, educational interventions are not typically designed to address the more complex psychological factors, including motivational issues, lack of confidence in vaccines, or distorted risk perceptions, that may underlie vaccine hesitancy.

Motivational interviewing and motivational communication (MI/MC) have emerged as behavioral counseling approaches used by HCPs to support patients in changing key health behaviors [10, 11]. Among the key behaviors targeted by MI/MC approaches is vaccine hesitancy, which has received increasing attention from health and behavioral scientists in recent years [12, 13]. MI is defined as “…a particular way of talking with people about behaviour change and growth to strengthen their own motivation and commitment to change” [14, 15], and MC, which was designed to be used by HCPs in the context of disease management, is defined as “... an evidence-based, time-efficient communication style used by healthcare providers to promote patient engagement, adoption of healthy behaviors, and sustained self-management of chronic conditions. It is informed by the behavioral sciences and emphasizes shared decision-making that is tailored to patients’ preferences, goals and values” [10]. The use of MI/MC styles essentially focuses on strengthening internal motivation (perceived importance and desire) and self-efficacy (perceived ability and confidence) to adopt a particular health behavior (e.g. getting a vaccine), while respecting patients’ current level of readiness [16]. Several systematic reviews and meta-analyses have shown that using MI/MC approach has been associated with an increased likelihood of adopting new, health behaviors such as drinking or smoking cessation, physical activity, and medication adherence among patients expressing ambivalence toward behavioral change [11, 17–20]. However, relatively few studies have examined the efficacy of MI/MC approaches to increase vaccine uptake. To our knowledge, no review has reported the relative efficacy of MI/MC and educational interventions on vaccination uptake.

This systematic review and meta-analysis aimed to compare the efficacy of MI/MC interventions and educational interventions on vaccine uptake relative to each other and compared with usual/standard of care. To achieve this objective, two sets of analyses were conducted. First, using all available data, vaccination rates were calculated for both interventions and then compared across interventions. Second, randomized controlled trial (RCT)-derived data were used to compare usual/standard of care standardized vaccination rates to educational and MI/MC interventions. We hypothesized that the efficacy of MI/MC interventions on vaccination rate would be superior to educational interventions.

## Methods

This systematic review was conducted and reported according to the Preferred Reporting Items for Systematic Reviews and Meta-Analyses (PRISMA guidelines) to ensure a thorough reporting of elements [17, 18]. The protocol of the systematic review was registered in the International Prospective Register of Systematic Reviews (PROSPERO: CRD42019140255).

### Search strategy

A systematic literature search was conducted in September 2021 and updated in November 2022, covering all related literature in three databases (PubMed, PsycINFO, and Cochrane trials). The keywords entered in the search engines were predefined based on their relevance to the objective of this review. They corresponded to key concepts related to vaccine hesitancy, behavioral interventions, and educational interventions (see supplementary files for exact search terms). No filters or publication date limits were applied.

### Study selection and screening

#### Inclusion/exclusion criteria

Studies aiming at improving vaccination rates using an educational and/or MI/MC intervention were included in this review. “Educational” interventions referred to interventions seeking to increase knowledge or awareness about vaccines (i.e. its benefits, the risks of not being vaccinated, and its safety) using different delivery formats (e.g. face-to-face consultations, using paper materials, or electronic mediums such as online information or access to videos). “MI/MC” interventions referred to interventions seeking to increase internal motivation for getting vaccinated by taking into consideration patients’ personal health goals and objectives and how their current behavior (e.g. refusing vaccination) may be misaligned with those objectives. To be included, the terminology “motivational interviewing/communication” or “educational intervention” must have been reported in the description of the intervention arms. Quasi-experimental, experimental, and observational study designs were included in the review, whereas other designs were excluded (e.g. case studies, reviews, and meta-analyses). For quasi-experimental and experimental designs, any comparator group was accepted. These could consist either of usual care, standard of care, or other types of interventions (reminders, lotteries, incentives) or a waiting list. Usual care refers to various types of care delivered across multiple settings, whereas standard of care refers to a type of uniform or standardized type of practice (often best practice) [21]. Eligible populations targeted by the intervention included adults (e.g. healthy adults, adults with any medical condition, parents/caregivers). Included studies also had to report vaccination rates pre- and post-intervention. The included studies were not limited to a specific type of vaccine. Multicomponent interventions, defined as any intervention delivered using multiple behavior change techniques in parallel, other than education (as a component) were excluded. Articles written in languages other than English or French were also excluded.

#### Screening procedure

One reviewer screened all titles and abstracts retrieved from the literature search (S.L.). In addition, titles and abstracts were divided and independently revised by five reviewers (V.G.B., B.V., L.A., N.T., and F.D.). Piloting was conducted using a standard set of 50 studies to ensure a consistent understanding of inclusion and exclusion criteria for identified studies. The primary reviewer (S.L.) and other reviewers discussed and resolved disagreements during the piloting.

Relevant articles were identified, and five authors independently assessed full texts (F.D., B.V., V.G.B., L.H.A., and N.T.). The agreement between S.L. and each reviewer was above 99% for the five dyads. Disagreements regarding the decision to include an article were resolved by discussion with a third reviewer (K.L.L.).

### Data extraction

In addition to the main reviewer (S.L.), three independent reviewers extracted the data from the selected articles (A.G., L.H.A., and N.T.). Piloting was conducted among 10 studies to ensure a consistent understanding of the extraction form, and any disagreements during piloting extraction were resolved through discussion. A predefined extraction form was used to perform the qualitative and quantitative data extraction. Qualitative data consisted of the following information: study design, type of comparator (if any), population targeted by the intervention, vaccine type, intervention (format, location), country, fidelity measure (if any), incentives (if any), and how vaccination rates were measured (e.g. self-reported, chart review). Quantitative data extracted consisted of outcomes (i.e. vaccination rates pre- and post-intervention, risk ratio with 95% CI, and *P*-value) and the number of participants in the intervention and comparator/control group. The average agreement among the three dyads of reviewers for the quantitative data was 88.7% (ranging from 86.5% to 90.3%). An additional reviewer adjudicated the data if an agreement still needed to be reached regarding the data extraction (K.L.L.).

### Study quality assessment

Study quality and risk of bias were assessed using the Downs and Black checklist by three independent reviewers organized into two dyads (S.L. and A.G., and S.L. and B.V., data available upon request) [22]. The Downs and Black checklist yielded 27 outcomes from 4 domains: (1) quality of reporting, (2) external validity, (3) internal validity, and (4) statistical power. The average agreement was 96.3% (i.e. 94.2% and 98.4% for the two dyads). All discrepancies of agreement on quality assessment were resolved through consultation and discussion between reviewers.

### Statistical analyses

Meta-analyses and risk of publication bias were conducted using Comprehensive Meta-Analysis (CMA, version 2.2) software. For the pre- to post-intervention analyses, we pooled the vaccination rates of each included study using a mixed-effects model (a random-effects model within subgroups and a fixed-effect model across subgroups) to compare educational interventions to MI/MC interventions. Pooled pre–post vaccination rates were calculated using a random-effects model for comparison groups (usual and standard of care) in order to obtain an absolute change value. For each RCT, we pooled the risk ratios of interventions (either education or MI/MC) compared with the comparison group (usual/standard of care) on post-intervention vaccination rate using a random-effects model, and each risk ratio extracted from studies needed to be accompanied by a corresponding standard error (SE). The SE was always derived from the confidence intervals provided.

We explored and quantified statistical heterogeneity between studies using the *I*^2^ statistic and chi-squared test, with 95% uncertainty intervals [22]. Subgroup analysis and meta-regression were used to explore sources of heterogeneity. Subgroup analyses were performed to explore the effect of both intervention types on vaccination rates according to the type of population (i.e. vaccine targeting adult-focused vaccination versus vaccine targeting children but given to parents) and according to intervention delivery format (face-to-face versus not face-to-face). The potential impact of study quality, using the Downs and Black score, on pooled post-intervention uptake was examined using meta-regression. A *P*-value of <.05 indicated significance in our analysis.

## Results

**Figure 1:**
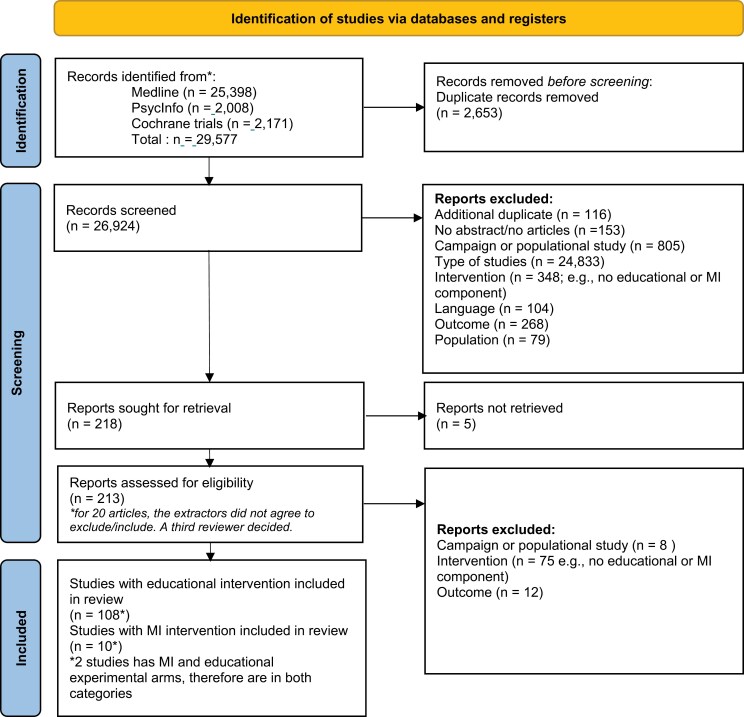
Preferred reporting items for systematic reviews and meta-analysis flow diagram

The literature search identified 26 924 articles that underwent title and abstract screening (Fig. 1). The authors of four studies [19, 20, 23, 24] were contacted to obtain unreported data. The data requested for two of these studies were not received, so these studies were excluded. This resulted in the identification of 213 potentially eligible studies. After a full-text review, 118 studies met the inclusion criteria: 108 educational and 10 MI/MC interventions. Two studies used MI interventions with an educational intervention comparator [25, 26]. Interventions (educational or MI/MC) were provided to unvaccinated participants; therefore, vaccination rates pre-intervention were 0% for all studies.

### Characteristics of educational intervention studies

Study characteristics of the 108 that reported on educational interventions are presented in Table S1. There were 64 RCTs and 44 quasi-experimental or pre–post studies. Half (*n* = 54) of the studies were conducted in the USA. Educational interventions were delivered to 95 980 participants across 176 experimental arms. In 73 (67.59%) studies, the intervention targeted adults and 35 (32.41%) studies targeted parents/caregivers of children. The influenza vaccine was targeted for 51 (47.22%) studies. Other targeted vaccines were Tuberculosis (BCG) (*n* =1, <1%), Diphtheria (*n* =1, <1%), Hepatitis (*n*=8, 7.41%), Herpes zoster (*n*=1, <1%), Human Papillomavirus (HPV) (*n*=23, 21.30%), Infant immunization (e.g. varicella, rotavirus, Tetanus-Diphtheria-Pertussis 3 doses (DPT3)) (*n*=16, 14.81%), Meningococcal (*n*=2, 1.85%), Measles-Mumps-Rubella (MMR) (*n*=2, 1.85%), Pneumococcal (*n*=11, 10.19%), Tetanus-Diphtheria-Pertussis (TDAP) (*n*=8, 7.41%), and COVID-19 (*n*=2, 1.85%). Nearly half of the educational studies (*n*=52, 48.15%) were conducted in the USA.

Multiple mediums were used to deliver education about vaccines to targeted populations. Half of the studies (*n*=55, 50.93%) used print materials to provide education (pamphlets, information letters), whereas face-to-face approaches were used in 47 (43.1%) studies, which were delivered mainly by HCPs (*n* = 33). Electronic (email, texts, mobile apps, video) formats were used in 33 (30.3%) studies. There were 30 (27.5%) studies that combined multiple delivery formats (e.g. face-to-face education, educational pamphlet, electronic format). A minority of studies (*n*=26, 23.9%) reported measuring the extent to which participants actually received the intervention and/or understood the information provided. Specifically, four studies asked participants if they received the intervention; eight studies used direct observation methods to determine receipt of the intervention; 5 studies recorded the number of visits or time spent on educational websites; and 10 studies asked participants questions about the information delivered.

### Characteristics of MI/MC intervention studies

Study characteristics for MI/MC interventions are presented in Table S2. Among the 10 MI/MC studies, 6 (60%) were RCTs, and 4 were quasi-randomized or pre–post studies. Most MI/MC studies (8/10) were conducted in the USA or Canada. Studies using MI/MC interventions were provided to a total of 9932 participants across 11 experimental arms. The interventions were delivered to adults (6/10, 60%) or parents/caregivers (4/10, 40%). Targeted vaccines were HPV (4/10, 40%), Influenza (2/10, 20%), Hepatitis (2/10, 20%), and routine immunization for children (e.g. varicella, rotavirus, Tetanus-Diphtheria-Pertussis 3 doses (DPT3)) (2/10, 20%). The MI/MC interventions were provided by HCPs (4/10, 40%), members of the research team (4/10, 40%), pharmacists (1/10, 10%), or administered online (1/10, 10%). One study provided vaccine education using a fact sheet about vaccines in addition to the face-to-face MI/MC [27]. Except for one study, all individuals who delivered MI/MC received training in MI/MC [27]. However, descriptions of the quantity and the quality of the training provided greatly varied across studies. Three studies issued brief statements that the person delivering MI/MC to participants received *standardized training in MI*, without providing details in terms of content, dose/duration, or if/how post-training competency was assessed [12, 28, 29]. Another study mentioned that MI/MC counseling was provided by a therapist *specialized in MI*, without providing details on how “being specialized” was defined or measured [25]. Five studies provided none or limited information about the type of MI/MC training received (i.e. content, time, type of feedback provided) [12, 23, 25, 27, 29]. A single study reported formally assessing whether MI/MC interventionists achieved competency in MI/MC according to standardized measures (e.g. Motivational Interviewing Treatment Integrity Code) [13, 30]. Regarding fidelity assessment of the MI/MC counseling delivered, one study reported the average time of each MI/MC counseling session [27], and one study monitored the MI counseling to ensure it was provided according to the treatment guide [25]. One study stated that “20% of the MI counseling sessions were monitored to ensure treatment fidelity,” but no details about the results of that exercise were provided [28]. None of the studies included other fidelity measures to ensure that the MI/MC interventions were delivered as intended.

### Description of comparison arms (RCTs only)

Comparison groups of RCTs consisted of usual care (*n* = 56), standard of care (*n*=8), reminders (*n* = 1), educational interventions (*n* = 7), and MI/MC (*n* = 1). Regarding the usual care group, descriptions provided by authors varied; the majority (*n* = 32) of RCTs provided short descriptions (i.e. immunization available on site, regular information about vaccines, routine care, general health information). In contrast, 19 RCTs did not provide any description. Four RCTs referring to “standard of care” provided a short description (i.e. general health education, routine medical visit, standard information about vaccine), and four did not provide a description but explicitly used the terms ¨standard of care¨.

### Pooled effect size of pre- to post-intervention efficacy for educational and MI/MC interventions

The effect size of vaccine uptake rate (as a percentage) pre- to post-intervention was calculated for educational and MI/MC intervention studies. The pooled effect size of the post-educational intervention of 177 arms was 0.52 (95% CI: 0.48–0.56), which means, on average, roughly 52% of the population got vaccinated after receiving an educational intervention. Heterogeneity was high between these studies (*Q* = 17 535, *df* = 176, and *P* < .001; *I*^2^ = 99%; *T*^2^ = 1.09). The pooled estimate of 11 arms of the pre- to post-MI/MC intervention vaccine update rate was 0.45 (95% CI: 0.29–0.62) (Table 1), which means, on average, roughly 45% of the population got vaccinated after receiving an MI/MC intervention. Heterogeneity was high between these studies (*Q* = 163 134, *df* = 10, and *P* < .001; *I*^2^ = 99%; *T*^2^ = 1.33). A mixed-effects analysis showed no significant difference in the mean effect size of vaccine rates between educational and MI/MC interventions (*Q* = 0.659, *P* = .417).

**Table 1:** Meta-analyses of post-intervention prevalence of vaccine uptake and risk ratio of interventions compared with the control group

Outcomes	Group	Number of arms	Pooled effect size	Heterogeneity				Mixed-effects analysis	
				*Q*-value	*I* ^2^ (%)	*T* ^2^	*P*-value	*Q*-value	*P*-value
Post-intervention prevalence of vaccine uptake	Education	177	0.52 (95% CI: 0.48–0.56)	17 535.84	99	1.09	<.001	0.659	.417
	MI or MC	11	0.45 (95% CI: 0.29–0.62)	1631.34	99	1.33	<.001		
	Control (education)	75	0.39 (95% CI: 0.34–0.45)	5289.02	99	1.10	<.001	–	–
	Control (MI or MC)	4	0.56 (95% CI: 0.34–0.76)	746.54	99	0.89	<.001		
Risk ratio of interventions compared with the control group	Education	93	1.10 (95% CI: 1.03–1.16)	774.30	84	0.084	<.001	0.031	.861
	MI or MC	5	1.07 (95% CI: 0.78–1.45)	25.37	99	1.09	<.001		

Notes:

• MI or MC refers to motivational interviewing/motivational communication.

• Control (education) refers to usual/standard of care groups as the comparison group for the education group.

• Control (MI or MC) refers to usual/standard of care groups as the comparison group for MI or MC group.

• The subgroup “adult” refers to interventions delivered solely to participants directly targeted by the vaccine (i.e. to adults rather than to parents/caregivers responsible for vaccinating children).

• The subgroup “caregiver” refers to an intervention delivered to parents/caregivers (and a vaccine targeted to their child).

#### Subgroup analysis

The effect size of the vaccination rates after receiving educational interventions was pooled for interventions delivered solely to participants directly targeted by the vaccine (i.e. to adults rather than to parents/caregivers responsible for vaccinating children), which included 119 arms. The pooled post-intervention effect was 0.44 (95% CI: 0.40–0.49) (Table 2). Similar analyses were conducted to calculate the pooled effect size of the vaccination rates for interventions delivered to parents/caregivers in the context of child vaccination. This included 58 arms for educational interventions, with a pooled effect size of 0.68 (95% CI: 0.61–0.75) (Table 2). Analyses revealed significant differences between the subgroups (*Q* = 28.13, *P* < .001) in favor of interventions delivered to parents/caregivers in the context of children immunization compared with participants directly targeted by the vaccine (i.e. adults vaccination).

**Table 2:** Subgroup analyses of post-intervention prevalence of vaccine uptake of interventions according to the type of population

Outcomes	Group	Subgroup	Number of arms	Pooled effect size	Heterogeneity				Mixed-effects analysis	
					*Q*-value	*I* ^2^ (%)	*T* ^2^	*P*-value	*Q*-value	*P*-value
Post-intervention prevalence of vaccine uptake	Education	Adult	119	0.44 (95% CI: 0.40–0.49)	10 812.50	99	0.98	<.001	28.13	<.001
		Caregiver	58	0.68 (95% CI: 0.61–0.75)	6538.91	99	1.53	<.001		
	MI or MC	Adult	7	0.28 (95% CI: 0.12–0.53)	578.62	99	1.99	<.001	12.19	<.001
		Caregiver	4	0.73 (95% CI: 0.67–0.78)	24.51	88	0.057	<.001		

Notes:

• MI or MC refers to motivational interviewing/motivational communication.

• The subgroup “adult” refers to interventions delivered solely to participants directly targeted by the vaccine (i.e. to adults rather than to parents/caregivers responsible for vaccinating children).

• The subgroup “caregiver” refers to an intervention delivered to parents/caregivers (and a vaccine targeted to their child).

Subgroup analyses were also conducted for MI/MC interventions. For the subgroup targeting adults, the pooled effect size of vaccine uptake was 0.28 (95% CI: 0.12–0.53) (Table 2). For the subgroup of parents/caregivers, the mean effect size of the pooled vaccination rate post-intervention was 0.73 (95% CI: 0.67–0.78) (Table 2). A significant difference between subgroups was identified (*Q* =12.19, *P* < .001) in favor of interventions delivered to parents/caregivers in the context of children immunization compared with participants directly targeted by the vaccine (i.e. adult immunization). The pooled post-intervention effect was calculated by delivery format (face-to-face [48 arms] versus not face-to-face, i.e. using paper or electrics modes). The pooled effect for face-to-face delivery formats was 0.63 (95% CI: 0.56–0.69). Similar analyses were conducted to calculate the pooled effect size for paper formats (71 arms). The pooled effect size for paper formats was 0.44 (95% CI: 0.38–0.51). Analyses revealed significant differences between these two subgroups (*Q* = 14.02, *P* < .001) in favor of face-to-face formats. The results are summarized in Table 3.

**Table 3:** Subgroup analyses of post-intervention prevalence of vaccine uptake of interventions according to the type of delivery format

Outcomes	Group	Subgroup	Number of arms	Pooled effect size	Mixed-effects analysis	
					*Q*-value	*P*-value
Post-intervention prevalence of vaccine uptake	Education	Face-to-face format	48	0.63 (95% CI: 0.56–0.69)	14.02	<.001
		Other format	71	0.44 (95% CI: 0.38–0.51)		

Notes:

• MI or MC refers to motivational interviewing/motivational communication.

• Other formats include paper formats, electronic, video, powerpoint.

#### Meta-regression

Meta-regression was conducted to analyze the impact of study quality on vaccination rates post-interventions. For the MI/MC intervention studies, the meta-regression using a random-effects model showed a significant positive effect for study quality on post-intervention vaccination rates (*F* = 5.55, *df* = (1,9), and *P* = .043), meaning that as quality increased there was a larger effect size. For educational intervention studies, the study quality meta-regression was not statistically significant (*F* = 2.68, *df* = (1,175), and *P* = .104).

### Effectiveness of educational and MI/MC interventions compared with usual/standard of care

There were 55 RCTs assessing educational interventions included in the meta-analysis. There were 93 arms comparing educational interventions with usual care/standard of care that were included in the meta-analysis. A random-effects model showed a small but significant effect for educational interventions compared with usual care (*RR* = 1.10; 95% *CI* = 1.03–1.16, *P* = .002) (Table 1). Of note, the pooled estimate for the vaccination rate of the 75 usual or standard of care comparison groups was 0.39 (95% CI: 0.34–0.45) (Table 1). There were four MI/MC RCTs accounting for five arms. Meta-analyses revealed no significant effect of MI/MC interventions on vaccination rates compared with usual or standard of care (*RR* = 1.07; 95% CI = 0.78–1.45, *P* = .691) (Table 1). The pooled effect size of the four usual/standard arms was 0.56 (95% CI: 0.34–0.76) (Table 1). Overall, mixed-effects analysis showed no significant difference in the risk ratio between education and MC/MI (*Q* = 0.299, *P* = .585).

## Discussion

This systematic review compared the efficacy of 10 MI/MC intervention studies versus 108 educational intervention studies on adult vaccination rates. The included studies were heterogeneous since they targeted multiple types of vaccines, populations, and formats of delivering the intervention. Comparisons were also conducted between each intervention type and usual/standard of care. Results revealed that pooled effect sizes showed 52% and 45% increases in vaccination rates for educational and MI/MC interventions, respectively, with no significant differences between the two. Further, analyses of the 55 RCTs using educational interventions and the four RCTs using MI/MC interventions showed that education was associated with a 10% increased likelihood of getting vaccinated compared with usual/standard of care. In contrast, MI/MC had no better effect on vaccination rates compared with usual care/standard of care.

Contrary to our expectations, the results of this review failed to observe any statistical superiority for MI/MC relative to the usual/standard of care. However, the mean *absolute* change in vaccination rate was larger for MI/MC (60%) than for educational (43%) interventions. Several previous studies have reported positive effects of MI/MC relative to usual/standard of care, which have found MI/MC approaches to be effective for changing multiple health behaviors, including smoking, alcohol consumption, physical activity, and medication adherence [11, 17–20]. However, in our review, the vaccination rate in the comparison groups of studies assessing the efficacy of MI/MC was higher (56%) than in the comparison groups of studies assessing the efficacy of educational interventions (39%). This could have reduced the potential to detect statistically significant effects of MI/MC. Comparison groups consisted either of “standard of care” or usual care. Standard of care is usually less subject to contamination due to its higher threshold of care relative to “usual care” comparators [21]. In fact, half (2/4) of the comparison groups in the MI/MC trials were categorized as “standard of care” when compared with only 11% (8/75) in the educational trial. Therefore, the type of comparator in the educational and MI/MC trials was not similar and highlights the importance of reporting fidelity for comparison groups in order to assess the potential for contamination. Unfortunately, fidelity measures were poorly reported in the studies identified in this review. In addition, only four RCTs included in the present meta-analysis used MI/MC approaches, which could have underpowered our comparisons and might have also contributed to our lack of significant results.

The significant but small efficacy of educational interventions on vaccination rates (relative to usual/standard of care) is consistent with the results of previous reviews. Dubé *et al*. systematically reviewed 15 reviews that documented the efficacy of various types of interventions (excluding MI/MC approaches) to address vaccine hesitancy or increase vaccine uptake [7] and concluded that the evidence for the efficacy of educational interventions was indeed small and inconsistent. In 2000, another extensive systematic review was conducted to determine the relative efficacy of different types of interventions, such as educational (again, excluding MI/MC) on vaccine uptake [31]. Interestingly, the authors found that interventions involving reminders to healthcare providers or patients effectively increased vaccine uptake, whereas education-only interventions did not. This suggests that education alone may be insufficient to increase vaccine uptake [8, 31].

Given the high heterogeneity between studies included in this review, we only had sufficient power to conduct two subgroup analyses, which were to compare (i) intervention efficacy between individual adults targeted by the vaccine and parents/caregivers with a child targeted by the vaccine and (ii) intervention delivery format within educational interventions (face-to-face versus other types). Both MI/MC and educational interventions targeting parents/caregivers were found to have a larger effect (effect size of 0.73 and 0.68 for MI/MC studies and educational studies, respectively, for child vaccine uptake) compared with those targeting individuals directly (effect size of 0.44 for educational studies and 0.28 for MI/MC studies). The significant relative efficacy of educational intervention to address parental vaccine hesitancy in the context of child vaccine uptake is consistent with a Cochrane review [32], which found a moderate effect in favor of face-to-face educational interventions on vaccine uptake among children (RR=1.20). Although parental and adult vaccine hesitancy share similar underlying determinants, such as a lack of confidence in health institutions and professionals, little is known about the factors underlying parental vaccine hesitancy, specifically. In a Canadian survey, parents identified the fear of being the “cause” of their child’s experience of side effects as one of the main reasons underlying parental vaccine hesitancy [33]. Our results align with reviews and nonclinical studies suggesting that both educational and MI/MC interventions may be especially effective in parental vaccine hesitancy for increasing rates of child vaccination [12, 32, 34]. The second subgroup analysis focused solely on educational interventions and compared the efficacy of face-to-face versus other delivery formats. The results revealed significantly stronger effects when education was delivered face to face. This is consistent with the benefits of two-way exchange and discussion, rather than one-way information delivery that encourages passivity, as is the case with most paper or electronic delivery formats [15]. Behavior change requires patient engagement rather than passivity, and this mechanism is difficult to trigger when receiving information sheets or passively watching an information video [15].

Education interventions are designed to increase vaccine uptake by increasing awareness and knowledge of vaccines and their benefits relative to their potential harms. In contrast, MI/MC interventions, which also provide information and challenge distorted beliefs, are designed to strengthen intrinsic motivation (perceptions of the importance of vaccines) and self-efficacy (confidence in the ability to get the vaccine) for vaccination, while also taking patient readiness for change into account [15, 35]. Moreover, MI/MC relies on using precise communication competencies to elicit patient motivation and skills, such as asking open questions and reflective listening, with constant adjustment of communication skills according to patient responses (i.e. change or resistance talk) [15]. This approach requires MI/MC training, practice, and feedback to achieve competence for those wishing to apply it effectively in practice. Although this requires a certain time investment, training courses can be relatively short (a few hours) and given in virtual formats, limiting the drawbacks of this approach [36, 37].

The fact that our review and several previous reviews revealed small but significant effects of educational interventions on vaccine uptake indicates that educational interventions could be part of a larger strategy to increase vaccination coverage. On the other hand, the relatively weak effect could be explained by the potentially counterproductive effects of providing vaccine education to a specific type of patients, such as those who are resistant (rather than just hesitant) to get vaccinated or those suffering from psychological reactance (extreme resistance of perceived threats to autonomy or freedom) [38, 39]. Educational interventions are delivered in a standardized way, and some patients may perceive these interventions as providing “unsolicited information or advice” that has been shown to strengthen rather than weaken resistance [14, 15]. For example, in 2015, an intervention was designed to address irrational beliefs about influenza vaccines being able to cause the flu [40]. A pre–post survey indicated that after receiving corrective information, participants’ beliefs changed, but in the opposite direction as intended: their intentions to receive the vaccine *decreased* rather than increased. Moreover, participants also reported having *more concerns* about the potential side effects of the vaccine after receiving the educational intervention than they had before. These results indicate the potential perverse effects of interventions that focus solely on providing general vaccine information that may be poorly tailored to the individual’s reasons for being vaccine-hesitant. These results also highlight the importance of targeting the right population for intervention, e.g. targeting patients at a specific stage of change or patients who are hesitant rather than resistant.

### Limitations of the literature

Articles retrieved from the literature search have limitations that should be considered when interpreting the results of this systematic review. First, comparison groups of RCTs were, for the most part, poorly described. Most studies provided only short or no descriptions, creating concerns about the potential presence of confounding variables and making it impossible to know if the studies were designed to provide a valid test, especially for MI/MC efficacy [34]. This is especially crucial for MI/MC counseling since it required communication skills, contrary to providing education that relies more on facts and can be delivered in a unidirectional way. Second, our results suggesting that study quality was correlated with efficacy only for MI/MC studies supports the need for better development and reporting of MI/MC interventions. For example, descriptions of the MI/MC training provided to interventionists in MI/MC trials were missing or only partially reported in 70% of the MI/MC studies, leading to uncertainty regarding the expertise and qualifications of the team. Thirdly, the included studies did not report change in the context of the patient’s stage of change, which could have provided more data on the effect of interventions on the motivational process underlying behavior change. Finally, few studies reported the results of fidelity assessments of the intervention (educational or MI/MC). Consequently, it is impossible to verify that the interventions were delivered as intended. In fact, the lack of a significant effect for MI/MC might be explained by poor study design (i.e. failure of the trial) rather than a lack of MI/MC efficacy (i.e. failure of the intervention).

### Limitations and strengths of the review

The high heterogeneity of the studies included in this review warrants caution when interpreting the results. The elevated level of heterogeneity is explained by the fact that any type of vaccine for any disease, and different study designs were included in the review, which limited the ability to conduct sensitivity analyses. Variability in how outcomes were reported should also be taken into account. For example, for studies measuring HPV vaccination rates (*n* = 23), some authors reported the outcome as the administration of all three doses (*n* = 3) and others reported it as the administration of only the first dose (*n* = 20). Also, some studies, even if they involved using the core skills of MI/MC, might not have been included because they did not fit the initial PICO (Population, Intervention, Control, Outcome) requirement that *authors must describe their intervention by referring to it as MI/MC* [37]. Since many did not adequately report on the content and theory behind their interventions, it was possible that some actual MI/MC interventions were missed. Adjustments in the search strategy should be considered for a future review. For example, the PICO inclusion criteria regarding the explicit mention of *Motivational Interviewing* could be replaced by terms referring to MI’s core concepts and skills (e.g. strengthening motivation and confidence, rolling with resistance, asking open questions, reflective listening) [15]. However, the lack of specificity of this kind of search would be prohibitive for this kind of review. In addition, a direct comparison (i.e. within the same study) of MI/MC versus educational interventions was not possible because of the small sample size (*k*=2). In fact, only two studies (RCTs) directly compared both types of interventions [25, 26]. Finally, both funnel plots for MI/MC RCTs and educational studies RCT revealed the presence of risk of bias (see Figures S4 and S5).

Despite some weaknesses, this study also has a number of important strengths. To our knowledge, this is the first review to synthesize data on the relative impact of traditional, educational interventions and motivational (MI/MC) interventions and their individual effects compared with usual or standard of care. This review conducted an extensive literature search, with multiple reviewers at all phases of screening data extraction, and quality assessment, consistent with PRISMA guidelines. The interjudge agreement was high. Finally, this work also highlights the crucial importance and need to develop and test behavioral interventions with rigor, including the importance of ensuring interventionist qualifications and assessing intervention and comparator fidelity.

## Conclusion

In conclusion, this systematic review reveals comparable efficacy of MI/MC (45%) approaches and educational interventions (52%) on vaccination rates. There was a significant, but small (10%) relative increase in the efficacy of educational interventions compared with usual/standard of care. In absolute terms, both types of interventions were better than usual/standard of care, with no statistical difference between the interventions. The most striking finding of this review is the small effect of educational interventions on vaccination rates. More research is needed to understand what type of patients this traditional approach seems to benefit (e.g. those already convinced of the vaccine benefits) and what type of patients it could be *counterproductive* in (e.g. patients being vaccine-hesitant). The overall poor quality of the studies contributes to low confidence in the results. Specifically, the failure to find evidence of the efficacy of MI/MC interventions on vaccine uptake was also unexpected, but likely attributable to a lack of power, high efficacy for comparator arms, high study heterogeneity, poor MI/MC studies quality, and a lack of reported fidelity measures. Future studies are needed to assess the efficacy of appropriately implemented and evaluated MI/MC approaches to improve vaccination rates, which have demonstrated efficacy in changing many other key health behaviors.

## Supplementary Material

ibae069_suppl_Supplementary_Tables_S1-S2_Figures_S3-S6

## Welfare of Animals

This article does not contain any studies with animals performed by any of the authors.

## Transparency Statement

The study was preregistered at the International Prospective Register of Systematic Reviews (PROSPERO) # CRD42019140255, https://www.crd.york.ac.uk/prospero/display_record.php?RecordID=140255. The analysis plan was not formally preregistered. Data from this study are not available in a public archive. Data from this study will be made available (as allowable according to institutional IRB standards) by emailing the corresponding author. There is no analytic code associated with this study. Materials used to conduct the study are not publicly available.
