# Supplementary material for: Addressing vaccine hesitancy: A systematic review comparing the efficacy of motivational versus educational interventions on vaccination uptake
Source: Transl Behav Med. 2025 Mar 26;15(1):ibae069. doi: 10.1093/tbm/ibae069 (PMC11942781; doi:10.1093/tbm/ibae069)
Supplement: ibae069_suppl_Supplementary_Tables_S1-S2_Figures_S3-S6 [file ibae069_suppl_supplementary_tables_s1-s2_figures_s3-s6.docx]

**Supplementary files**

**S1.**

**Table 1. Characteristics of educational interventions**

| **First author (year, country)** | **Study design** | **Arm (format)** | **Vaccine target** | **Professional delivering Education (when delivered by a person)** | **Population receiving the intervention (education)*** | **n** | **Post-intervention vaccination rate** | **Included in meta-analysis** |
| --- | --- | --- | --- | --- | --- | --- | --- | --- |
| Abramson, Z. H. (2010, Israel)**(1) | RCT | Education (Face-to-face) | Influenza | Family Physicians | HCPs | 163 | 52.8% | Yes |
|  |  | Control (Usual/control care: immunization available on the site) |  |  |  | 181 | 26.5% |  |
| Abu-rish, E.Y. (2020, Jordan)(2) | Non-RCT | Education (Paper) | Pneumococcal |  | Elderly adults | 683 | 1.9% | No |
| Altay, M. (2016, Turkey)(3) | Non-RCT | Education (Face-to-face) | Influenza | Unknown- not reported | Diabetic patients | 579 | 36.6% | No |
|  |  | Education (Face-to-face) | Pneumococcus | Unknown- not reported | Diabetic patients | 579 | 12.4% |  |
|  |  | Education (Face-to-face) | Hepatitis B | Unknown- not reported | Diabetic patients | 579 | 3.8% |  |
| Ami, S. (2019, Israel)(4) | Non-RCT | Education (Face-to-face) | Influenza | Physicians | Elderly adults | 53 | 51% | No |
| Anjum, Q. (2004, India)(5) | RCT | Education (Face-to-face) | BCG | Medical student | Parents/Caregivers | 189 | 93% | Yes |
|  |  | Education (Face-to-face) | OPV 3 | Medical student | Parents/Caregivers | 180 | 84% |  |
|  |  | Education (Face-to-face) | DPT 3 | Medical student | Parents/Caregivers | 180 | 77% |  |
|  |  | Education (Face-to-face) | Measles | Medical student | Parents/Caregivers | 160 | 74% |  |
|  |  | Control (Usual care; no intervention) | BCG |  | Parents/Caregivers | 164 | 81% |  |
|  |  | Control (Usual care; no intervention) | OPV 3 |  | Parents/Caregivers | 159 | 65% |  |
|  |  | Control (Usual care; no intervention) | DPT 3 |  | Parents/Caregivers | 159 | 56% |  |
|  |  | Control (Usual care; no intervention) | Measles |  | Parents/Caregivers | 136 | 58% |  |
| Aragones, A. (2015, US)(6) | Non-RCT | Education (face-to-face) | HPV | Trained workers | Parents/Caregivers | 24 | 87% | No |
|  |  | Education (face-to-face); reminder | HPV | Trained workers | Parents/Caregivers | 45 | 98% |  |
| Armstrong, K. (1999, US)**(7) | RCT | Education (Paper) | Influenza |  | Elderly adults | 390 | 66.4% | No |
|  |  | Control (Reminder) | Influenza |  | Elderly adults | 350 | 56.9% |  |
| Baltes,M (2022, US)(8) | Non-RCT | Education (Phamphlet) | Influenza |  | Adults | 658 | 60.9% | No |
| Bennett, T.A. (2014, US)(9) | RCT | Education (email) | HPV |  | Adults | 332 | 7.8% | Yes |
|  |  | Control (Usual\control care – usual and general website about vaccines) | HPV |  | Adults | 332 | 8.7% |  |
| Bernard-Genest, MP. (2021, Canada)(10) | RCT | Education (Paper and telephone) | HPV |  | Adults | 62 | 15.4% | No |
|  |  | Education (Paper) | HPV |  | Adults | 68 | 11.7% |  |
| Bernstein, H. H. (2017, US)**(11) | Non-RCT | Education (Face-to-face; paper) | TDAP | Nurses | Adults | 632 | 73% | No |
| Bertin, M. (2007, US)**(12) | Non-RCT | Education (Electronic – Computer) | Influenza |  | HCPs | 17,998 | 55% | No |
| Bielecki, K. (2020, Scotland)(13) | Non-RCT | Education (Paper) | Influenza |  | Parents/Caregivers | 855 | 53.2% | No |
|  |  | Control (Usual care, i.e. no intervention delivered) | Influenza |  | Parents/Caregivers | 917 | 48.2% |  |
| Black, M.E. (1993, Canada)(14) | RCT | Education (Face-to-face) | Influenza | Nurses | Elderly adults | 157 | 56.1% | Yes |
|  |  | Control (Usual care; no more description) | Influenza |  | Elderly adults | 148 | 56.6% |  |
| Bloom, H.G. (1988, US)(15) | RCT | Education (paper, email, brochure) | Influenza |  | Elderly adults | 25 | 76.0% | No |
|  |  | Education (document and face-to-face) | Influenza | Nurse | Elderly adults | 25 | 80.0% |  |
|  |  | Education (document and face-to-face) | Influenza | Volunteer | Elderly adults | 23 | 78.2% |  |
|  |  | Education (paper, email, brochure) | Pneumococcal |  | Elderly adults | 20 | 75.0% |  |
|  |  | Education (document and face-to-face) | Pneumococcal | nurse | Elderly adults | 20 | 75.0% |  |
|  |  | Education (document and face-to-face) | Pneumoccocal | Volunteer | Elderly adults | 18 | 75.0% |  |
| Borg, K. (2018, Australia)(16) | RCT | Education (Personalized letter) | Influenza |  | Parents/Caregivers | 1845 | 5.9% | Yes |
|  |  | Education (Pamphlet) | Influenza |  | Parents/Caregivers | 1845 | 4.5% |  |
|  |  | Control (usual care, i.e. existing practice) |  |  | Parents/Caregivers | 1844 | 4.4% |  |
| Bryant, K. A. (2004, US)(17) | Non-RCT | Education (Paper) | Influenza |  | HCPs | 633 | 60% | No |
|  |  | Control (Usual care; i.e. no educational intervention provided) | Influenza |  | HCPs | 483 | 45% |  |
| Burger, MN. (2022, Germany)(18) | RCT | Education (email) | COVID |  | Adults | 205 | 67.8% | Yes |
|  |  | Education (email) | COVID |  | Adults | 207 | 73.4% |  |
|  |  | Control (reminder) | COVID |  | Adults | 189 | 66.1% |  |
|  |  | Control (standard of care; not descriped) | COVID |  | Adults | 220 | 64.1% |  |
| Bushar, J. A. (2017, US)**(19) | Non-RCT | Electronic (Mobile app.) | Influenza |  | Adults | 377 | 81.3% | No |
|  |  | Control (Usual care; participants who did not receive the intervention) | Influenza |  | Adults | 2824 | 47.1% |  |
| Carter, William B. (1986, US)(20) | RCT | Education (Paper) | Influenza |  | Elderly or patients with chronic illness | 121 | 36% | Yes |
|  |  | Control (Usual care, i.e. usual standard letter sent) | Influenza |  | Elderly or patients with chronic illness | 114 | 23% |  |
| Cassidy, B. (2014, US)**(21) | Non-RCT | Education (Pamphlet/brochure and face-to-face) | HPV | Pediatric resident | Parents/Caregivers | 24 | 75% | No |
| Cassidy, W.M. (1995, US)(22) | Non-RCT | Education (Paper and face-to-face) | Hepatitis B | Class teacher | Parents/Caregivers | 654 | 65% | No |
| Cebollero, J. (2020, US)(23) | Non-RCT | Education Face-to-face, paper) | HPV | Pharmacist | Adults | 80 | 35% | No |
| Chan, Sophia S. C. (2015, Hong Kong)(24) | RCT | Education (Face-to-face and telephone) | Pneumococcal | Nurses | Elderly adults with medical condition | 1,251 | 57.2% | Yes |
|  |  | Control (Standard care as reported by authors, i.e., leaflets, health education, posters) | Pneumococcal |  | Elderly adults with medical condition | 1,266 | 48.1% |  |
| Cheng, P. J. (2015, Taiwan)(25) | Non-RCT | Education (Face-to-face) | TDAP | Physicians | Parents/Caregivers | 3,186 | 71.2% | No |
| Coenen, S. (2017, Belgium)(26) | RCT | Education (Paper form and face-to-face) | Influenza | Nurses | Adults with chronic disease | 140 | 36% | Yes |
|  |  | Education (Paper form and face-to-face) | Pneumococcus | Nurses | Adults with chronic disease | 140 | 62% |  |
|  |  | Education (Paper form and face-to-face) | Hepatitis B | Nurses | Adults with chronic disease | 140 | 27% |  |
|  |  | Education (Paper form and face-to-face) | Tetanus | Nurses | Adults with chronic disease | 140 | 33% |  |
|  |  | Control (Standard care. i.e. standard explanation about vaccine) | Influenza |  | Patients with chronic disease | 206 | 10% |  |
|  |  | Control (Standard care i.e. standard explanation about vaccine) | Pneumococcus |  | Adults with chronic disease | 206 | 23% |  |
|  |  | Control (Standard care i.e. standard explanation about vaccine) | Hepatitis B |  | Adults with chronic disease | 206 | 5% |  |
|  |  | Control (Standard care i.e. standard explanation about vaccine) | Tetanus |  | Adults with chronic disease | 206 | 2% |  |
| Colon-Lopez, V. (2021, Puerto Rico)(27) | Non-RCT | Education (face to face) | HPV |  | Adults | 19 | 5.3 | No |
| Collins, L. (2003, US)**(28) | Non-RCT | Education (Paper) | Meningococcal |  | Adults | 1,562 | 46% | No |
| Costantino, C. (2019, Italy)(29) | Non-RCT | Education (video, seminar) | Influenza |  | HCPs | 38 | 42% | No |
|  |  | Control (Usual care, i.e., participant refusing to receive the intervention) | Influenza |  | HCPs | 87 | 31% |  |
| Costantino, C. (2021, Italy)a(30) | Non-RCT | Education (face to face) | Influenza | Public Health medical doctor | Adults | 201 | 47.8% | No |
|  |  | Education (face to face) | Tdap |  | Adults | 201 | 57.7% |  |
| Currat, M. (2020, Switzerland)(31) | RCT | Education (face-to-face) | Influenza | Nurses | HCPs | 185 | 50% | Yes |
|  |  | Control (Usual care, i.e. no description) | Influenza |  | HCPs | 172 | 46% |  |
| Deuson, R. R. (2001, US)**(32) | Non-RCT | Education (Face-to-face, paper and video) | Hepatitis B | Member of the community | Parents/Caregivers | 4,384 | 15.5% | No |
| Dixon, E.M (2019, US)(33) | RCT | Education (Web-based) | HPV |  | Parents/Caregivers | 141 | 78% | Yes |
|  |  | Control (Usual care; no more description) | HPV |  | Parents/Caregivers | 1,455 | 52.8% |  |
| Doratotaj, S. (2008, US)**(34) | RCT | Education (Paper) | Influenza |  | HCPs and clinic staff | 200 | 39% | Yes |
|  |  | Education (Paper); Raffle | Influenza |  | HCPs and clinic staff | 200 | 44.5% |  |
|  |  | Control (Raffle) | Influenza |  | HCPs and clinic staff | 200 | 42% |  |
|  |  | Control (Usual care, i.e. no additional intervention thant the usual: poster, newsletters, access to vaccine) | Influenza |  | HCPs and clinic staff | 200 | 38% |  |
| Drokow, EK. (2021, Ghana)(35) | Non-RCT | Education (video) | HPV |  | Adults | 600 | 32% | No |
| Elangovan, S. (1996, US)(36) | Non-RCT | Education (Paper form and face-to-face) | Pneumococcus | Unknown – not reported | Elderly adults | 244 | 79% | No |
| Ferson, M.J. (1995, Australia)(37) | RCT | Education (telephone) | Infant Immunization |  | Parents/Caregivers | 49 | 71% | No |
|  |  | Education (Paper) | Infant Immunization |  | Parents/Caregivers | 54 | 37% |  |
| Fuchs, J. (2006, Germany)(38) | Non-RCT | Education (Face-to-face) | Diphteria | Pharmacists | Adults | 312 | 71.2% | No |
|  |  | Education (Face-to-face) | Tetanus | Pharmacists | Adults | 312 | 86.5% |  |
|  |  | Education (Face-to-face) | Influenza | Pharmacists | Adults | 312 | 74.7% |  |
|  |  | Education (Face-to-face) | Pneumococcal | Pharmacists | Adults | 312 | 62.8% |  |
|  |  | Education (Face-to-face) | Encephalitis | Pharmacists | Adults | 312 | 42.4% |  |
| Ginson, S.H. (2000, Canada)(39) | RCT | Education (Face-to-face, paper) | Influenza | Pharmacists | Elderly adults | 28 | 61% | Yes |
|  |  | Control (Usual care; participants did not received the intervention) | Influenza |  | Elderly adults | 37 | 16% |  |
|  |  | Education (Face-to-face, paper) | Pneumococcal | Pharmacists | Elderly adults | 49 | 67% |  |
|  |  | Control (Usual care; participants did not received the intervention) | Pneumococcal |  | Elderly adults | 48 | 21% |  |
| Glanz, M.J. (2020, US)(40) | RCT | Education (web-based, personalized) | MMR |  | Adults | 194 | 94.84% | Yes |
|  |  | Education (web-based, not personalized) | MMR |  | Adults | 213 | 95.77% |  |
|  |  | Control (Usual care, i.e. standard vaccine information statement) | MMR |  | Adults | 183 | 93.99% |  |
|  |  | Education (web-based, personalized) | Varicella |  | Adults | 194 | 95.36% |  |
|  |  | Education (web-based, not personalized) | Varicella |  | Adults | 213 | 94.84% |  |
|  |  | Control (Usual care, i.e. standard vaccine information statement) | Varicella |  | Adults | 183 | 95.08% |  |
| Gołębiak, I. (2020, Poland)(41) | RCT | Education 1 (Paper and face-to-face) | Influenza | ¨ an educator¨ | HCPs | 80 | 36.2% | No |
|  |  | Education 1a(Paper and face-to-face) | Influenza | ¨ an educator¨ | HCPs | 82 | 18.2% |  |
|  |  | Education 2 (Paper) | Influenza |  | HCPs | 85 | 20.0% |  |
|  |  | Education 2a (Paper) | Influenza |  | HCPs | 82 | 17.1% |  |
| Goodman, K. (2015, US)(42) | RCT | Education (Video) | Influenza |  | Parents/Caregivers | 53 | 28% | Yes |
|  |  | Control (Usual care; i.e. participants watched video on another topic) | Influenza |  | Parents/Caregivers | 52 | 25% |  |
| Hohmeier, K.C. (2016, US)(43) | Non-RCT | Education (face-to-face) | HPV | Pharmacists | Adults | 21 | 47.6% | No |
| Hu, Y. (2017, China)(44) | RCT | Education (Face-to-face) | Infant immunization (TDP3) | Physicians | Parents/Caregivers | 418 | 90% | Yes |
|  |  | Control (Usual care, i.e., not educational intervention) | Infant immunization (TDP3) |  | Parents/Caregivers | 433 | 82.9% |  |
| Hu, Y. (2018, China)(45) | RCT | Education (Video) | Varicella |  | Parents/Caregivers | 68 | 86.4% | Yes |
|  |  | Education (Paper form) | Varicella |  | Parents/Caregivers | 68 | 76.1% |  |
|  |  | Control (Usual care; no description) | Varicella |  | Parents/Caregivers | 68 | 56.7% |  |
| Ho, J.H. (2019, Singapore)(46) | Non-RCT | Education (Paper) | Influenza |  | Elderly adults | 4,378 | 5.9% | No |
|  |  | Control (Usual care; no description) | Influenza |  | Elderly adults | 4,459 | 4.8% |  |
|  |  | Education (Paper) | Pneumoccocus |  | Elderly adults | 4,378 | 5.7% |  |
|  |  | Control (Usual care; no description) | Pneumoccocus |  | Elderly adults | 4,459 | 3.7% |  |
| Hu, PL. (2023, Singapore)(47) | RCT | Education (face-to-face) | Influenza | Team member | Elderly adults | 160 | 10% | No |
|  |  | Education (Phamphlet) | Influenza |  | Elderly adults | 160 | 12.5% |  |
| Jacobson, T. A. (1999, US)(48) | RCT | Education (Paper form) | Pneumococcus |  | Elderly and patients with medical condition | 163 | 19.9% | Yes |
|  |  | Control (Usual care, i.e. educational document on another topic) | Pneumococcus |  | Elderly and patients with medical condition | 155 | 3.8% |  |
| Jiang, M. (2022, China)(49) | RCT | Education (video) | Influenza |  | Elderly adults | 175 | 10.3% | Yes |
|  |  | Control (Usual care; i,e, basic medical exam only) | Influenza |  | Elderly adults | 175 | 3.4% |  |
| Jimenez-Quinones, E. M. (2017, Porto Rico)(50) | Non-RCT | Education (Paper form and face-to-face) | HPV | Pharmacists | Adults | 7 | 28.6% | No |
| Johnson, E. A. (2003, US)(51) | Non-RCT | Education (Online video and semainar); Paper; reminder | Pneumococcal |  | Adults | 114 | 12.3% | No |
|  |  | Control (reminder) | Pneumococcal |  | Adults | 108 | 10% |  |
| Jordan, E. T. (2015, US)(52) | RCT | Education (electronic texto) | Influenza |  | Mothers/caregivers | 1,025 | 21% | Yes |
|  |  | Control (Usual care; participants received usual message) | Influenza |  | Pregnant women | 1,228 | 22% |  |
|  |  | Education (electronic texto) | Influenza |  | Mothers/caregivers | 687 | 10% |  |
|  |  | Control (Usual care; participants received usual message) | Influenza |  | Pregnant women | 880 | 11% |  |
| Juraskova, I. (2011, Australia)(53) | RCT | Education (Paper about cervical cancer) | HPV |  | Adults | 39 | 33.3% | No |
|  |  | Education (Paper about cervical cancer and genital warts) | HPV |  | Adults | 36 | 41.7% |  |
| Kimura, A. C. (2007, US)**(54) | RCT | Education (Paper-form and video) | Influenza |  | HCPs | 821 | 34% | Yes |
|  |  | Education (Paper-form and video); Increase accessibility | Influenza |  | HCPs | 754 | 53% |  |
|  |  | Control (Increased accessibility) | Influenza |  | HCPs | 832 | 46% |  |
|  |  | Control (Usual care, i.e. participants did not receive interventions) | Influenza |  | HCPs | 1,517 | 28% |  |
| Klassing, H.M. (2017, US)(55) | RCT | Education (phone) | Influenza |  | Adults with medical condition | 77 | 72.7% | Yes |
|  |  | Education (paper, email, brochure) | Influenza |  | Adults with medical condition | 63 | 83.7% |  |
|  |  | Control (Usual care, i.e. no intervention) | Influenza |  | Adults with medical condition | 70 | 88.6% |  |
|  |  | Education (phone) | Pneumoccocal |  | Adults with medical condition | 77 | 59.7% |  |
|  |  | Education (paper, email, brochure) | Pneumoccocal |  | Adults with medical condition | 63 | 61.9% |  |
|  |  | Control (Usual care, i.e. no intervention) | Pneumoccocal |  | Adults with medical condition | 70 | 55.7% |  |
| Krieger, J.W. (2000, US)(56) | RCT | Education (Paper and telephone) | Pneumoccocal |  | Elderly adults | 327 | 51.9% | Yes |
|  |  | Control (Usual care, i.e., newsletter, pamphlets, posters) | Pneumoccocal |  | Elderly adults | 112 | 30.9% |  |
|  |  | Education (Paper and telephone) | Influenza |  | Elderly adults | 622 | 88.2% |  |
|  |  | Control (Usual care, i.e., newsletter, pamphlets, posters) | Influenza |  | Elderly adults | 624 | 81.7% |  |
| Kriss, J. L. (2017, US)(57) | RCT | Education (Video) | TDAP |  | Pregnant women | 31 | 29% | Yes |
|  |  | Education (Electronic interactive tutorial) | TDAP |  | Pregnant women | 30 | 50% |  |
|  |  | Control (Usual care, i.e., standard CDC vaccine statement not targeted to pregnant women) | TDAP |  | Pregnant women | 34 | 18% |  |
| Kuntz, J. L. (2008, US)**(58) | Non-RCT | Education (Face-to-face) | Influenza | Nurses | HCPs | 5,467 | 66% | No |
| Lee, Hee Yun (2016, US)(59) | Non-RCT | Education (Electronic texto) | HPV |  | Adults | 30 | 30% | No |
| Lee, (2021, US)(60) | RCT | Education (web-based) | Influenza |  | Adults | 5,158 | 62.2% | Yes |
|  |  | Control (Usual care, no description) | Influenza |  | Adults | 5,271 | 61.1% |  |
| Leung, K. C. (2017, Hong Kong)(61) | RCT | Education (Pamphlet; Face-to-face) | Influenza | Investigator | Elderly adults | 265 | 33.6% | Yes |
|  |  | Control (Usual care, i.e., usual poster and leaflets but no intervention) | Influenza |  | Elderly adults | 264 | 25% |  |
| Lubis, TA. (2022, Indonesia)(62) | Non-RCT | Education (video) | Infant immunization |  | Parents/Caregivers | 116 | 79.3% | No |
|  |  | Control (Usual care, i.e. acces to general health care document) | Infant immunization |  | Parents/Caregivers | 104 | 72.1% |  |
| Lv, K. (2021, China)(63) | RCT | Education (internet based) | Infant immunization |  | Parents/Caregivers | 49 | 81.6% | Yes |
|  |  | Control (Usual care, i.e. routine nursing) | Infant immunization |  | Parents/Caregivers | 49 | 63.27% |  |
| Meharry, P.M. (2014, US)(64) | RCT | Education (Paper) | Influenza |  | Parents/Caregivers | 48 | 72.9% | Yes |
|  |  | Education (Paper; face-to-face) | Influenza | Unknown – not reported | Parents/Caregivers | 36 | 86.1% |  |
|  |  | Control (Usual care, i.e., no direct vaccine information) | Influenza |  | Parents/Caregivers | 49 | 46.9% |  |
| Moran, P.W. (1996, US)(65) | RCT | Education (paper) | Influenza |  | Adults with medical condition | 198 | 36% | Yes |
|  |  | Education (paper); incentive | Influenza |  | Adults with medical condition | 199 | 26% |  |
|  |  | Control (Usual care, no more description) | Influenza |  | Adults with medical condition | 202 | 20% |  |
|  |  | Control (Incentive) | Influenza |  | Adults with medical condition | 198 | 29% |  |
| Munoz-Miralles, R. (2022, Spain)(66) | RCT | Education (face to face; Paper) | Influenza | HCPs | Adults with medical condition | 251 | 29.9% | Yes |
|  |  | Control (Usual care, i.e., usual advice) | Influenza |  | Adults with medical condition | 273 | 14.6% |  |
| Nyamathi, A. (2010, US)****(67) | RCT | Education (face to face) | Hepatitis | Nurses | Adults | 55 | 39% | No |
|  |  | MI (individual) | Hepatitis |  | Adults | 50 | 29% |  |
|  |  | MI (group) | Hepatitis |  | Adults | 43 | 31% |  |
| Oeffinger, K. C. (1992, US)(68) | RCT | Education (Paper; Face-to-face) | Infant immunization | family practice resident | Parents/Caregivers | 116 | 31% | Yes |
|  |  | Control (Usual care, i.e., no special education about vaccine) | Infant immunization |  | Parents/Caregivers | 122 | 33% |  |
| O'Leary, S.T. (2019, US)(69) | RCT | Education (web-based) (social media) | Influenza |  | Adults | 140 | 57.14% | Yes |
|  |  | Education (web-based) | Influenza |  | Adults | 105 | 56.19% |  |
|  |  | Control (Usual care, i.e., no more description) | Influenza |  | Adults | 44 | 36.36% |  |
|  |  | Education (web-based)(social media) | Tdap |  | Adults | 80 | 71.25% |  |
|  |  | Education (web-based) | Tdap |  | Adults | 62 | 69.35% |  |
|  |  | Control (Usual care, i.e., no more description) | Tdap |  | Adults | 31 | 67.74% |  |
| Orgul., G. (2021, Turkey)(70) | Non-RCT | Education (face to face) | Influenza | Experienced cliniciens | Adults | 353 | 54.1% | No |
| Otsuka, S. H. (2013, US)(71) | RCT | Education (Paper and outcome measured via personal health) | Herpes Zoster |  | Elderly adults | 250 | 13.2% | Yes |
|  |  | Control (standard of care as reported by authors and outcome measured via personal health) | Herpes Zoster |  | Elderly adults | 424 | 5% |  |
|  |  | Education (Paper and outcome measured via non-personal health) | Herpes Zoster |  | Elderly adults | 250 | 5.2% |  |
|  |  | Control (Standard care as reported by authors and outcome measured via non-personal health) | Herpes Zoster |  | Elderly adults | 1,665 | 1.8% |  |
| Otsuka-Ono, H. (2019, Japan)(72) | RCT | Education (face-to-face) + Education (paper, email, brochure) | Hepatitis B | paediatrician or pharmacist | Parents/Caregivers | 87 | 76% | Yes |
|  |  | Control (Usual care, i.e. no description) | Hepatitis B |  | Parents/Caregivers | 84 | 49% |  |
|  |  | Education (face-to-face) + Education (paper, email, brochure) | Rotavirus | paediatrician or pharmacist | Parents/Caregivers | 87 | 84% |  |
|  |  | Control (Usual care, i.e. no description) | Rotavirus |  | Parents/Caregivers | 84 | 68% |  |
|  |  | Education (face-to-face) + Education (paper, email, brochure) | Influenza | paediatrician or pharmacist | Parents/Caregivers | 87 | 95% |  |
|  |  | Control (Usual care, i.e. no description) | Influenza |  | Parents/Caregivers | 84 | 95% |  |
|  |  | Education (face-to-face) + Education (paper, email, brochure) | Pneumoccocal | paediatrician or pharmacist | Parents/Caregivers | 87 | 97% |  |
|  |  | Control (Usual care, i.e. no description) | Pneumoccocal |  | Parents/Caregivers | 84 | 94% |  |
| Owais, A. (2011, Pakistan)(73) | RCT | Education (Paper) | Hepatitis B |  | Parents/Caregivers | 179 | 72.1% | Yes |
|  |  | Control (Usual care, i.e., general health promotion messages) | Hepatitis B |  | Parents/Caregivers | 178 | 51.7% |  |
| Payaprom, Y. (2011, Thailand)(74) | RCT | Education (Paper) | Influenza |  | Patients with chronic condition | 99 | 89% | Yes |
|  |  | Control (Usual care, i.e., standard government leaflet) | Influenza |  | Patients with chronic condition | 102 | 86% |  |
| Piedimonte, S. (2018, Canada)(75) | Non-RCT | Education (face-to-face) | HPV | Physicians | Adults | 151 | 42% | No |
| Powell-Jackson, C.T. (2018, India)(76) | RCT | Education (Paper positive frame) | TDAP |  | Parents/caregivers | 232 | 41% | Yes |
|  |  | Education (Paper, negative frame) | TDAP |  | Parents/caregivers | 239 | 45% |  |
|  |  | Control (Usual care, i.e., no information given to participants) | TDAP |  | Parents/caregivers | 235 | 28% |  |
| Reiter, P.L. (2018, US)(77) | RCT | Education (Paper); Reminder | HPV |  | Adults | 76 | 45% | Yes |
|  |  | Control (Usual care, i.e., standard information about HPV) | HPV |  | Adults | 74 | 26% |  |
| Richman, A. R. (2016, US)***(78) | RCT | Education (Electronic messages) | HPV |  | Adults | 130 | 53% | Yes |
|  |  | Control (Standard care as describe by auhors) | HPV |  | Adults | 134 | 52% |  |
| Rodriguez, R.M (1993, US)(79) | Non-RCT | Education (Paper) | Influenza |  | Elderly adults | 84 | 50% | No |
|  |  | Education (Paper) | Pneumococcal |  | Elderly adults | 109 | 57.8% |  |
| Rothan-Tondeur, M. (2010, France)(80) | RCT | Education (Electronic, Video) | Influenza |  | HCPs | 1,201 | 34% | Yes |
|  |  | Control (Usual care, i.e., no action performed) | Influenza |  | HCPs | 1,144 | 32% |  |
| Saitoh, A. (2013, Japan)(81) | RCT | Education (Face-to-face) | Influenza | Investigator | Parents/Caregivers | 70 | 95.7% | Yes |
|  |  | Control (Usual care, i.e. routine check-up visits) | Influenza |  | Parents/Caregivers | 36 | 88.9% |  |
|  |  | Education (Face-to-face) | Pneumococcus | Investigator | Parents/Caregivers | 70 | 94.3% |  |
|  |  | Control (Usual care, i.e. routine check-up visits) | Pneumococcus |  | Parents/Caregivers | 36 | 88.9% |  |
|  |  | Education (Face-to-face) | Hepatitis B | Investigator | Parents/Caregivers | 70 | 34.3% |  |
|  |  | Control (Usual care, i.e. routine check-up visits) | Hepatitis B |  | Parents/Caregivers | 36 | 11.1% |  |
| Saitoh, A. (2017, Japan)(82) | RCT | Education (Face-to-face) | Infant immunization | Midwifes | Parents/Caregivers | 100 | 43% | Yes |
|  |  | Control (usual care, i.e., pamphlet during routine check-up but no oral intervention) | Infant immunization |  | Parents/Caregivers | 88 | 45.5% |  |
| Saitoh, A. (2020, Japan)(83) | Non-RCT | Education (Paper) | Rotavirus |  | Parents/Caregivers | 111 | 91% | No |
|  |  | Control (Usual care, i.e., no intervention) | Rotavirus |  | Parents/Caregivers | 119 | 99% |  |
|  |  | Education (Paper) | Hepatitis B |  | Parents/Caregivers | 111 | 97% |  |
|  |  | Control (Usual care, i.e., no intervention) | Hepatitis B |  | Parents/Caregivers | 119 | 99% |  |
|  |  | Education (Paper) | Influenza |  | Parents/Caregivers | 111 | 97% |  |
|  |  | Control (Usual care, i.e., no intervention) | Influenza |  | Parents/Caregivers | 119 | 99% |  |
|  |  | Education (Paper) | Pneumoccocal |  | Parents/Caregivers | 111 | 97% |  |
|  |  | Control (Usual care, i.e., no intervention) | Pneumoccocal |  | Parents/Caregivers | 119 | 99% |  |
| Sanderson, M. (2017, US)(84) | Non-RCT | Education (Face-to-face, Paper and video) | HPV | Nurses or Medical Assistant | Parents/Caregivers | 167 | 45.4% | No |
|  |  | Control (Usual care, i.e., no video presented) | HPV |  | Parents/Caregivers | 138 | 32.9% |  |
| Schluter, W. W. (1999, US)**(85) | Non-RCT | Education (Paper and video) | Influenza |  | Elderly adults | 595 | 89% | No |
| Scott, V.P (2019, US)(86) | RCT | Education (paper, email, brochure - national data) | Influenza |  | Adults | 134 | 73.1% | Yes |
|  |  | Education (paper, email, brochure - local data) | Influenza |  | Adults | 133 | 76.7% |  |
|  |  | Control (Usual care, i.e., no more description by authors) | Influenza |  | Adults | 133 | 65.4% |  |
| Shegog, R. (2022, US)(87) | RCT | Education (app) | HPV |  | Parents/Caregivers | 98 | 49% | Yes |
|  |  | Control (Usual care, i.e., participants no access to intervention app) | HPV |  | Parents/Caregivers | 207 | 47.7% |  |
| Si., M. (2022, China)(88) | RCT | Education (internet-based) | HPV |  | Adults | 1582 | 2.2% | Yes |
|  |  | Usual care; information on another topic | HPV |  | Adults | 1489 | 1.8% |  |
| Stille, C. J. (2001, US)(89) | RCT | Education (Paper and face-to-face) | Infant immunization | ¨Provider¨ | Parents/Caregivers | 156 | 58.3% | Yes |
|  |  | Control (Usual care, i.e., routine information about immunizations) | Infant immunization |  | Parents/Caregivers | 159 | 57.9% |  |
| Tapiainen, T. (2005, Switzerland)**(90) | Non-RCT | Education (Paper and face-to-face) | Influenza | Nurses | HCPs | 554 | 24% | No |
| Takamatsu, A. (2022, Japan)(91) | Non-RCT | Education (multifacet) | COVID |  | HCPs | 1224 | 92.1% | No |
| Thomas, D.M. (2003, US)(92) | RCT | Education (Paper; video) | Pneumoccocal |  | Adults | 165 | 23.3% | Yes |
|  |  | Education (Video) | Pneumoccocal |  | Adults | 162 | 10.2% |  |
|  |  | Control (Usual care; general brochure about health) | Pneumoccocal |  | Adults | 145 | 6.6% |  |
| Thomas, C.M. (2005, Canada)(93) | Non-RCT | Education (face-to-face, paper) | Pneumococcal | Resident, staff nurse and nurse coordinator | Adults with medical condition | 50 | 54% | No |
|  |  | Control (Usual care, i.e., usual advice) | Pneumococcal |  | Patients with medical condition | 38 | 0% |  |
| Tisi, G. (2013, Italy)***(94) | Non-RCT | Education (Paper) | HPV |  | Parents/Caregivers | 161 | 44% | No |
| Tubeuf, S. (2014, England)(95) | RCT | Education (Paper) | MMR |  | Parents/Caregivers | 85 | 91% | Yes |
|  |  | Control (Decision aid) | MMR |  | Parents/Caregivers | 48 | 100% |  |
|  |  | Control (Usual care, i.e., usual service provided) | MMR |  | Parents/Caregivers | 70 | 99% |  |
| Underwood, N. L. (2019, US)(96) | RCT | Education (Paper) | TDAP |  | Parents/Caregivers | 668 | 24.7% | Yes |
|  |  | Education (Paper) | TDAP |  | Parents/Caregivers | 290 | 27.3% |  |
|  |  | Education (Paper) | Meningococcal |  | Parents/Caregivers | 668 | 35.2% |  |
|  |  | Education (Paper) | Meningococcal |  | Parents/Caregivers | 690 | 37% |  |
|  |  | Education (Paper to parents only) | HPV |  | Parents/Caregivers | 668 | 27% |  |
|  |  | Education (Paper and teens) | HPV |  | Parents/Caregivers | 690 | 24.4% |  |
|  |  | Control (Usual care, i.e. no information was sent to participants) | HPV |  | Parents/Caregivers | 777 | 26.1% |  |
|  |  | Control (Usual care, i.e. no information was sent to participants ) | TDAP |  | Parents/Caregivers | 777 | 34.5% |  |
|  |  | Control (Usual care, i.e. no information was sent to participants) | Meningococcal |  | Parents/Caregivers | 777 | 37.7% |  |
| Usami, T. (2009, Japan)(97) | RCT | Education (Paper, face-to-face) | Influenza | Pharmacists | Elderly adults | 36 clusters (n=911) | 81.6% | Yes |
|  |  | Control (Usual care, i.e., no discussion about immunization) | Influenza |  | Elderly adults | 43 clusters (n=952) | 64.9% |  |
| Usman, R.H. (2009, Pakistan)(98) | RCT | Education (Face-to-face) | TDAP | Trained data collector | Parents/Caregivers | 375 | 65% | Yes |
|  |  | Education (Face-to-face; reminder) | TDAP | Trained data collector | Parents/Caregivers | 375 | 74% |  |
|  |  | Control (Reminder) | TDAP |  | Parents/Caregivers | 375 | 69% |  |
|  |  | Control (Standard care. i.e, no interventions and routine visit) | TDAP |  | Parents/Caregivers | 375 | 55% |  |
| Usman, R.H. (2011, Pakistan)(99) | RCT | Education (Face-to-face) | DPT3 | Unknown – not reported | Parents/Caregivers | 376 | 61% | Yes |
|  |  | Control (Reminder) | DPT3 |  | Parents/Caregivers | 378 | 67% |  |
|  |  | Control (Standard care. i.e, routine visit) | DPT3 |  | Parents/Caregivers | 376 | 39% |  |
| Van Amburgh, J. A. (2001, US)(100) | Non-RCT | Education (Paper) | Influenza |  | Adults | 657 | 53.9% | No |
| Wang, Z. (2021, China)****(101) | RCT | MI | HPV |  | Adults | 208 | 17.3% | No |
|  |  | Education (website) | HPV |  | Adults | 208 | 11.5% |  |
|  |  | Education (website with tutorial) | HPV |  | Adults | 208 | 7.2% |  |
| Watson, B. (1998, US)(102) | Non-RCT | Education (Paper and face-to-face) | Hepatitis | ¨Workers¨ | Parents/Caregivers | 3,099 | 34% | No |
| Weaver, F. M. (2007,US)(103) | Non-RCT | Education (Paper) | Influenza |  | Patients with medical condition | 2,214 | 67,4% | No |
| Webb, N.J. (2022, US)(104) | Non-RCT | Education (Face to face) | Influenza | Dermatologists | Adults | 85 | 84.7 | No |
| Wedel, S. (2016, US)(105) | Non-RCT | Education (Face-to-face) | HPV | Nurses | Adults | 103 | 91% | No |
| Williams, S. E. (2013, US)(106) | RCT | Education (Video, paper) | Infant immunization |  | Parents/Caregivers | 55 | 83% | Yes |
|  |  | Control (Usual care, i.e., routine care) | Infant immunization |  | Parents/Caregivers | 67 | 81.8% |  |
| Winer, R. L. (2016, US)***(107) | RCT | Education (Video, power point) | HPV |  | Parents/Caregivers | 25 | 32% | Yes |
|  |  | Control (Usual care. i.e. presentation of diabete information) | HPV |  | Parents/Caregivers | 29 | 28% |  |
| Wong, V. W. Y. (2016, Hong Kong)(108) | RCT | Education (Face-to-face) | Influenza | Nurses | Parents/Caregivers | 161 | 22,5% | Yes |
|  |  | Control (Standard care. i.e., routine checkup and general health education) | Influenza |  | Parents/Caregivers | 160 | 10% |  |

**Note:**

RCT = Randomized controlled trial**;** HCPs = Healthcare providers; BCG = Bacillus Calmette-Guerin, a vaccine for tuberculosis disease; OPV 3 = oral poliovirus vaccine; DPT 3 =  [diphtheria](https://en.wikipedia.org/wiki/Diphtheria), [pertussis](https://en.wikipedia.org/wiki/Pertussis)(whooping cough), and [tetanus](https://en.wikipedia.org/wiki/Tetanus); TDAP = Tetanus, Diphtheria, Pertussis; Infant immunization =

Vaccination rate for HPV correspond to the reception of at least 1 vaccine and not completion (i.e. 3 vaccines).

*n correspond to number of participants with post-intervention data

**Not explicitly mentioned by the authors that intervention was delivered to non-vaccinated participants

***Rate correspond to completion of HPV immunization consisting of the uptake of 3 vaccines. Rate of initiation only (1 or 2 vaccine received) was not reported.

**** Study also included in MI analysis

**S2.**

**Table 2. Characteristics of motivational interviewing/communication (MI/MC) intervention studies**

| **First author (year country)** | **Study design** | **Arm (format)** | **Vaccine target** | **Professional delivering MI/MC** | **Population receiving the intervention (MI counseling)** | **n** | **Post-intervention vaccination rate** | **Included in meta-analysis** |
| --- | --- | --- | --- | --- | --- | --- | --- | --- |
| Berenbrok, LA., (2022, US)(109) | Non-RCT | Experimental, MI | Hepatitis | Pharmacist | Adults | 1569 | 3.9% | No |
|  |  | Standard care |  | NA |  | 3640 | 0% |  |
| Jamison, KC. (2022, US)(110) | Non-RCT | Experimental, MI | Infant immunization | Trained HCP | Parents/caregivers | 66 | 87.9% | No |
| Joseph, N. P. (2016, US)(111) | RCT | Experimental, MI | HPV | Trained research assistant | Parents/caregivers | 98 | 56% | Yes |
|  |  | Standard practice as stated by authors; no intervention |  | NA |  | 102 | 51% |  |
| Labbé, S., (2022, Canada)(112) | Non-RCT | Experimental, MI | Influenza | Trained HCP | Adults | 15 | 33% | No |
| Nyamathi, A. (2010, US)***(67) | RCT | Experimental: MI individual | Hepatitis | Trained therapist | Adults with medical condition | 50 | 29% | No |
|  |  | Experimental: MI group |  |  |  | 43 | 31% |  |
|  |  | Education face-to-face, non-MI 55 |  |  |  | 55 | 39% |  |
| Real, F. J. (2017, US)(113) | RCT | Experimental MI | Influenza | Trained physician | Adults | 253 | 67,5% | Yes |
|  |  | Standard care as stated by authors with standard information about influenza |  |  |  | 235 | 60% |  |
| Gagneur, A. (2018,Canada)(114) | Non-RCT | Experimental MI | Infant immunization | Trained research assistant | Parents/caregivers | 1128 | 75.9% | No |
|  |  | Standard care |  |  |  | 1249 | 68.6% |  |
| Wang, Z. (2021, China)**,***(101) | RCT | Experimental MI | HPV | Trained Fieldwork | Adults | 208 | 17.3% | No |
|  |  | Education on website |  |  |  | 208 | 11.5% |  |
|  |  | Education on tutorial website |  |  |  | 208 | 7.2% |  |
| Dempsey, A.F. (2018,US)*(115) | RCT | Experimental MI | HPV | Trained HCP | Adults | 2507 | 42.9% | Yes |
|  |  | Usual care; usual discussion about vaccine, not intervention |  |  |  | 2747 | 38.9% |  |
| Pot, M. (2017, Netherlands)(116) | RCT | MI (Electronic, interactive) | HPV | Web-based | Parents/Caregivers | 3995 | 73.32% | Yes |
|  |  | Control (Usual care; regular information about vaccine) | HPV |  |  | 4067 | 72.81% |  |

Note:

HCP = Healthcare Providers; HPV = Human Papillomavirus; MI = Motivation Interviewing; RCT = Randomized Controlled Trial

*Rate correspond to completion of HPV immunization consisting of the uptake of 3 vaccines. Rate of initiation only (1 or 2 vaccine received) was not reported.

** Rate correspond to HPV completion. No data reported for HPV initiation (1, 2 doses).

*** Arm with educational intervention also included in table 2.

**S2.**

**Table 2. Characteristics of motivational interviewing/communication (MI/MC) intervention studies**

| **First author (year country)** | **Study design** | **Arm (format)** | **Vaccine target** | **Professional delivering MI/MC** | **Population receiving the intervention (MI counseling)** | **n** | **Post-intervention vaccination rate** | **Included in meta-analysis** |
| --- | --- | --- | --- | --- | --- | --- | --- | --- |
| Berenbrok, LA., (2022, US)(109) | Non-RCT | Experimental, MI | Hepatitis | Pharmacist | Adults | 1569 | 3.9% | No |
|  |  | Standard care |  | NA |  | 3640 | 0% |  |
| Jamison, KC. (2022, US)(110) | Non-RCT | Experimental, MI | Infant immunization | Trained HCP | Parents/caregivers | 66 | 87.9% | No |
| Joseph, N. P. (2016, US)(111) | RCT | Experimental, MI | HPV | Trained research assistant | Parents/caregivers | 98 | 56% | Yes |
|  |  | Standard practice as stated by authors; no intervention |  | NA |  | 102 | 51% |  |
| Labbé, S., (2022, Canada)(112) | Non-RCT | Experimental, MI | Influenza | Trained HCP | Adults | 15 | 33% | No |
| Nyamathi, A. (2010, US)***(67) | RCT | Experimental: MI individual | Hepatitis | Trained therapist | Adults with medical condition | 50 | 29% | No |
|  |  | Experimental: MI group |  |  |  | 43 | 31% |  |
|  |  | Education face-to-face, non-MI 55 |  |  |  | 55 | 39% |  |
| Real, F. J. (2017, US)(113) | RCT | Experimental MI | Influenza | Trained physician | Adults | 253 | 67,5% | Yes |
|  |  | Standard care as stated by authors with standard information about influenza |  |  |  | 235 | 60% |  |
| Gagneur, A. (2018,Canada)(114) | Non-RCT | Experimental MI | Infant immunization | Trained research assistant | Parents/caregivers | 1128 | 75.9% | No |
|  |  | Standard care |  |  |  | 1249 | 68.6% |  |
| Wang, Z. (2021, China)**,***(101) | RCT | Experimental MI | HPV | Trained Fieldwork | Adults | 208 | 17.3% | No |
|  |  | Education on website |  |  |  | 208 | 11.5% |  |
|  |  | Education on tutorial website |  |  |  | 208 | 7.2% |  |
| Dempsey, A.F. (2018,US)*(115) | RCT | Experimental MI | HPV | Trained HCP | Adults | 2507 | 42.9% | Yes |
|  |  | Usual care; usual discussion about vaccine, not intervention |  |  |  | 2747 | 38.9% |  |
| Pot, M. (2017, Netherlands)(116) | RCT | MI (Electronic, interactive) | HPV | Web-based | Parents/Caregivers | 3995 | 73.32% | Yes |
|  |  | Control (Usual care; regular information about vaccine) | HPV |  |  | 4067 | 72.81% |  |

Note:

HCP = Healthcare Providers; HPV = Human Papillomavirus; MI = Motivation Interviewing; RCT = Randomized Controlled Trial

*Rate correspond to completion of HPV immunization consisting of the uptake of 3 vaccines. Rate of initiation only (1 or 2 vaccine received) was not reported.

** Rate correspond to HPV completion. No data reported for HPV initiation (1, 2 doses).

*** Arm with educational intervention also included in table 2.

References

41. Abramson ZH, Avni O, Levi O, Miskin IN. Randomized trial of a program to increase staff influenza vaccination in primary care clinics. Ann Fam Med. 2010;8(4):293-8.

42. Abu-Rish EY, Barakat NA. The impact of pharmacist-led educational intervention on pneumococcal vaccine awareness and acceptance among elderly in Jordan. Hum Vaccin Immunother. 2021;17(4):1181-9.

43. Altay M, Ates I, Altay FA, Kaplan M, Akca O, Ozkara A. Does education effect the rates of prophylactic vaccination in elderly diabetics? Diabetes Res Clin Pract. 2016;120:117-23.

44. Schattner A. Cardiovascular-targeted patient education and uptake of influenza vaccination in elderly patients. Patient Educ Couns. 2020;103(5):1052-4.

45. Anjum Q, Omair A, Inam SN, Ahmed Y, Usman Y, Shaikh S. Improving vaccination status of children under five through health education. J Pak Med Assoc. 2004;54(12):610-3.

46. Aragones A, Bruno DM, Ehrenberg M, Tonda-Salcedo J, Gany FM. Parental education and text messaging reminders as effective community based tools to increase HPV vaccination rates among Mexican American children. Prev Med Rep. 2015;2:554-8.

47. Armstrong K, Berlin M, Schwartz JS, Propert K, Ubel PA. Educational content and the effectiveness of influenza vaccination reminders. J Gen Intern Med. 1999;14(11):695-8.

48. Baltes M, Pronovost E, Kanuch S, Caron A. Improving Influenza Vaccination Rates in an Underserved Primary Care Clinic Using a Simple Educational Tool. Qual Manag Health Care. 2022;31(2):80-4.

49. Bennett AT, Patel DA, Carlos RC, Zochowski MK, Pennewell SM, Chi AM, et al. Human Papillomavirus Vaccine Uptake After a Tailored, Online Educational Intervention for Female University Students: A Randomized Controlled Trial. J Womens Health (Larchmt). 2015;24(11):950-7.

50. Bernard-Genest MP, Ruel-Laliberte J, Lapointe-Milot K. Effect of educative reminder telephone calls on human papillomavirus immunization rate: A randomized controlled trial. Womens Health (Lond). 2021;17:17455065211003821.

51. Bernstein HH, Monty M, Yang P, Cohen A. Increasing Tdap Coverage Among Postpartum Women: A Quality Improvement Intervention. Pediatrics. 2017;139(3).

52. Bertin M, Scarpelli M, Proctor AW, Sharp J, Robitson E, Donnelly T, et al. Novel use of the intranet to document health care personnel participation in a mandatory influenza vaccination reporting program. Am J Infect Control. 2007;35(1):33-7.

53. Bielecki K, Craig J, Willocks LJ, Pollock KG, Gorman DR. Impact of an influenza information pamphlet on vaccination uptake among Polish pupils in Edinburgh, Scotland and the role of social media in parental decision making. BMC Public Health. 2020;20(1):1381.

54. Black ME, Ploeg J, Walter SD, Hutchinson BG, Scott EA, Chambers LW. The impact of a public health nurse intervention on influenza vaccine acceptance. Am J Public Health. 1993;83(12):1751-3.

55. Bloom HG, Bloom JS, Krasnoff L, Frank AD. Increased utilization of influenza and pneumococcal vaccines in an elderly hospitalized population. J Am Geriatr Soc. 1988;36(10):897-901.

56. Borg K, Sutton K, Beasley M, Tull F, Faulkner N, Halliday J, et al. Communication-based interventions for increasing influenza vaccination rates among Aboriginal children: A randomised controlled trial. Vaccine. 2018;36(45):6790-5.

57. Bryant KA, Stover B, Cain L, Levine GL, Siegel J, Jarvis WR. Improving influenza immunization rates among healthcare workers caring for high-risk pediatric patients. Infect Control Hosp Epidemiol. 2004;25(11):912-7.

58. Bushar JA, Kendrick JS, Ding H, Black CL, Greby SM. Text4baby Influenza Messaging and Influenza Vaccination Among Pregnant Women. Am J Prev Med. 2017;53(6):845-53.

59. Carter WB, Beach LR, Inui TS. The flu shot study: using multiattribute utility theory to design a vaccination intervention. Organ Behav Hum Decis Process. 1986;38(3):378-91.

60. Cassidy B, Braxter B, Charron-Prochownik D, Schlenk EA. A quality improvement initiative to increase HPV vaccine rates using an educational and reminder strategy with parents of preteen girls. J Pediatr Health Care. 2014;28(2):155-64.

61. Cassidy WM, Mahoney FJ. A hepatitis B vaccination program targeting adolescents. J Adolesc Health. 1995;17(4):244-7.

62. Cebollero J, Walton SM, Cavendish L, Quairoli K, Cwiak C, Kottke MJ. Evaluation of Human Papillomavirus Vaccination After Pharmacist-Led Intervention: A Pilot Project in an Ambulatory Clinic at a Large Academic Urban Medical Center. Public Health Rep. 2020;135(3):313-21.

63. Chan SS, Leung DY, Leung AY, Lam C, Hung I, Chu D, et al. A nurse-delivered brief health education intervention to improve pneumococcal vaccination rate among older patients with chronic diseases: a cluster randomized controlled trial. Int J Nurs Stud. 2015;52(1):317-24.

64. Cheng PJ, Huang SY, Su SY, Peng HH, Chang CL. Increasing postpartum rate of vaccination with tetanus, diphtheria, and acellular pertussis vaccine by incorporating pertussis cocooning information into prenatal education for group B streptococcus prevention. Vaccine. 2015;33(51):7225-31.

65. Coenen S, Weyts E, Jorissen C, De Munter P, Noman M, Ballet V, et al. Effects of Education and Information on Vaccination Behavior in Patients with Inflammatory Bowel Disease. Inflamm Bowel Dis. 2017;23(2):318-24.

66. Colon-Lopez V, Ayala-Marin A, Velez-Alamo C, Soto-Salgado M, Medina-Cortes L, Acevedo-Fontanez AI, et al. inverted exclamation markHabla de VPH! An Educational Activity for College Students in Puerto Rico. P R Health Sci J. 2021;40(3):142-6.

67. Collins L, Dupont L, Nagle D. The impact of educational efforts on first-year university students' acceptance of meningococcal vaccine. J Am Coll Health. 2003;52(1):41-3.

68. Costantino C, Restivo V, Gaglio V, Lanza GLM, Marotta C, Maida CM, et al. Effectiveness of an educational intervention on seasonal influenza vaccination campaign adherence among healthcare workers of the Palermo University Hospital, Italy. Ann Ig. 2019;31(1):35-44.

69. Costantino C, Mazzucco W, Bonaccorso N, Cimino L, Conforto A, Sciortino M, et al. Educational Interventions on Pregnancy Vaccinations during Childbirth Classes Improves Vaccine Coverages among Pregnant Women in Palermo's Province. Vaccines (Basel). 2021;9(12).

70. Currat M, Lazor-Blanchet C, Zanetti G. Promotion of the influenza vaccination to hospital staff during pre-employment health check: a prospective, randomised, controlled trial. J Occup Med Toxicol. 2020;15(1):34.

71. Deuson RR, Brodovicz KG, Barker L, Zhou F, Euler GL. Economic analysis of a child vaccination project among Asian Americans in Philadelphia, Pa. Arch Pediatr Adolesc Med. 2001;155(8):909-14.

72. Dixon BE, Zimet GD, Xiao S, Tu W, Lindsay B, Church A, et al. An Educational Intervention to Improve HPV Vaccination: A Cluster Randomized Trial. Pediatrics. 2019;143(1).

73. Doratotaj S, Macknin ML, Worley S. A novel approach to improve influenza vaccination rates among health care professionals: a prospective randomized controlled trial. Am J Infect Control. 2008;36(4):301-3.

74. Drokow EK, Effah CY, Agboyibor C, Sasu E, Amponsem-Boateng C, Akpabla GS, et al. The Impact of Video-Based Educational Interventions on Cervical Cancer, Pap Smear and HPV Vaccines. Front Public Health. 2021;9:681319.

75. Elangovan S, Kallail KJ, Vargo G. Improving pneumococcal vaccination rates in an elderly population by patient education in an outpatient clinic. J Am Board Fam Pract. 1996;9(6):411-3.

76. Ferson MJ, Fitzsimmons G, Christie D, Woollett H. School health nurse interventions to increase immunisation uptake in school entrants. Public Health. 1995;109(1):25-9.

77. Fuchs J. The provision of pharmaceutical advice improves patient vaccination status. Pharm Pract (Granada). 2006;4(4):163-7.

78. Ginson HS, Malmberg C, French JD. Impact on Vaccination Rates of a Pharmacist-Initiated Influenza and Pneumococcal Vaccination Program. The Canadian Journal of Hospital Pharmacy. 2000;53.

79. Glanz JM, Wagner NM, Narwaney KJ, Pyrzanowski J, Kwan BM, Sevick C, et al. Web-Based Tailored Messaging to Increase Vaccination: A Randomized Clinical Trial. Pediatrics. 2020;146(5).

80. Golebiak I, Okreglicka K, Kanecki K, Nitsch-Osuch A. The impact of selected educational and information interventions on the coverage rate and attitudes to influenza vaccination in nursing staff. Med Pr. 2020;71(6):665-85.

81. Goodman K, Mossad SB, Taksler GB, Emery J, Schramm S, Rothberg MB. Impact of Video Education on Influenza Vaccination in Pregnancy. J Reprod Med. 2015;60(11-12):471-9.

82. Hohmeier KC, Randolph DD, Smith CT, Hagemann TM. A multimodal approach to improving human papillomavirus vaccination in a community pharmacy setting. SAGE Open Med. 2016;4:2050312116682128.

83. Hu Y, Chen Y, Wang Y, Song Q, Li Q. Prenatal vaccination education intervention improves both the mothers' knowledge and children's vaccination coverage: Evidence from randomized controlled trial from eastern China. Hum Vaccin Immunother. 2017;13(6):1-8.

84. Hu Y, Li Q, Chen Y. Evaluation of two health education interventions to improve the varicella vaccination: a randomized controlled trial from a province in the east China. BMC Public Health. 2018;18(1):144.

85. Ho HJ, Tan YR, Cook AR, Koh G, Tham TY, Anwar E, et al. Increasing Influenza and Pneumococcal Vaccination Uptake in Seniors Using Point-of-Care Informational Interventions in Primary Care in Singapore: A Pragmatic, Cluster-Randomized Crossover Trial. Am J Public Health. 2019;109(12):1776-83.

86. Hu PL, Koh EYL, Tay JSH, Chan VX, Goh SSM, Wang SZ. Assessing the impact of educational methods on influenza vaccine uptake and patient knowledge and attitudes: a randomised controlled trial. Singapore Med J. 2023;64(2):98-104.

87. Jacobson TA, Thomas DM, Morton FJ, Offutt G, Shevlin J, Ray S. Use of a low-literacy patient education tool to enhance pneumococcal vaccination rates. A randomized controlled trial. JAMA. 1999;282(7):646-50.

88. Jiang M, Yao X, Li P, Fang Y, Feng L, Hayat K, et al. Impact of video-led educational intervention on uptake of influenza vaccine among the elderly in western China: a community-based randomized controlled trial. BMC Public Health. 2022;22(1):1128.

89. Jimenez-Quinones EM, Melin K, Jimenez-Ramirez FJ. Impact of a Pharmacist Conducted Educational Program on Human Papilloma Virus Vaccination Rates in a Low Socioeconomic Population in the City of Lares, PR. P R Health Sci J. 2017;36(2):67-70.

90. Johnson EA, Harwell TS, Donahue PM, Weisner MA, McInerney MJ, Holzman GS, et al. Promoting pneumococcal immunizations among rural Medicare beneficiaries using multiple strategies. J Rural Health. 2003;19(4):506-10.

91. Jordan ET, Bushar JA, Kendrick JS, Johnson P, Wang J. Encouraging Influenza Vaccination Among Text4baby Pregnant Women and Mothers. Am J Prev Med. 2015;49(4):563-72.

92. Juraskova I, Bari RA, O'Brien MT, McCaffery KJ. HPV vaccine promotion: does referring to both cervical cancer and genital warts affect intended and actual vaccination behavior? Womens Health Issues. 2011;21(1):71-9.

93. Kimura AC, Nguyen CN, Higa JI, Hurwitz EL, Vugia DJ. The effectiveness of vaccine day and educational interventions on influenza vaccine coverage among health care workers at long-term care facilities. Am J Public Health. 2007;97(4):684-90.

94. Klassing HM, Ruisinger JF, Prohaska ES, Melton BL. Evaluation of Pharmacist-Initiated Interventions on Vaccination Rates in Patients with Asthma or COPD. J Community Health. 2018;43(2):297-303.

95. Krieger JW, Castorina JS, Walls ML, Weaver MR, Ciske S. Increasing influenza and pneumococcal immunization rates: a randomized controlled study of a senior center-based intervention. Am J Prev Med. 2000;18(2):123-31.

96. Kriss JL, Frew PM, Cortes M, Malik FA, Chamberlain AT, Seib K, et al. Evaluation of two vaccine education interventions to improve pertussis vaccination among pregnant African American women: A randomized controlled trial. Vaccine. 2017;35(11):1551-8.

97. Kuntz JL, Holley S, Helms CM, Cavanaugh JE, Vande Berg J, Herwaldt LA, et al. Use of a pandemic preparedness drill to increase rates of influenza vaccination among healthcare workers. Infect Control Hosp Epidemiol. 2008;29(2):111-5.

98. Lee HY, Koopmeiners JS, McHugh J, Raveis VH, Ahluwalia JS. mHealth Pilot Study: Text Messaging Intervention to Promote HPV Vaccination. Am J Health Behav. 2016;40(1):67-76.

99. Lee JL, Foschini L, Kumar S, Juusola J, Liska J, Mercer M, et al. Digital intervention increases influenza vaccination rates for people with diabetes in a decentralized randomized trial. NPJ Digit Med. 2021;4(1):138.

100. Leung KC, Mui C, Chiu WY, Ng YY, Chen MHY, Ho PH, et al. Impact of patient education on influenza vaccine uptake among community-dwelling elderly: a randomized controlled trial. Health Educ Res. 2017;32(5):455-64.

101. Lubis TA, Gunardi H, Herqutanto, Soedjatmiko S, Satari HI, Alatas FS, et al. Educational videos to address vaccine hesitancy in childhood immunization. Vaccine. 2022;40(41):5965-70.

102. Lv K, Zhao J, Zhang P. The effect of community comprehensive nursing using scenario-based health education on the infant and young child immunization rates. Am J Transl Res. 2021;13(8):9663-70.

103. Meharry PM, Cusson RM, Stiller R, Vazquez M. Maternal influenza vaccination: evaluation of a patient-centered pamphlet designed to increase uptake in pregnancy. Matern Child Health J. 2014;18(5):1205-14.

104. Moran WP, Nelson K, Wofford JL, Velez R, Case LD. Increasing influenza immunization among high-risk patients: education or financial incentive? Am J Med. 1996;101(6):612-20.

105. Munoz-Miralles R, Bonvehi Nadeu S, Sant Masoliver C, Martin Gallego A, Gomez Del Canto J, Mendioroz Pena J, et al. Effectiveness of a brief intervention for acceptance of influenza vaccine in reluctant primary care patients. Gac Sanit. 2022;36(5):446-51.

106. Oeffinger KC, Roaten SP, Hitchcock MA, Oeffinger PK. The effect of patient education on pediatric immunization rates. J Fam Pract. 1992;35(3):288-93.

107. O'Leary ST, Narwaney KJ, Wagner NM, Kraus CR, Omer SB, Glanz JM. Efficacy of a Web-Based Intervention to Increase Uptake of Maternal Vaccines: An RCT. Am J Prev Med. 2019;57(4):e125-e33.

108. Orgul G, Keles AI, Yucel A, Sahin D. The Rate of Influenza Vaccination after Face-to-Face Interview in Pregnancy. Z Geburtshilfe Neonatol. 2021;225(5):423-7.

109. Otsuka SH, Tayal NH, Porter K, Embi PJ, Beatty SJ. Improving herpes zoster vaccination rates through use of a clinical pharmacist and a personal health record. Am J Med. 2013;126(9):832 e1-6.

110. Otsuka-Ono H, Hori N, Ohta H, Uemura Y, Kamibeppu K. A childhood immunization education program for parents delivered during late pregnancy and one-month postpartum: a randomized controlled trial. BMC Health Serv Res. 2019;19(1):798.

111. Owais A, Hanif B, Siddiqui AR, Agha A, Zaidi AK. Does improving maternal knowledge of vaccines impact infant immunization rates? A community-based randomized-controlled trial in Karachi, Pakistan. BMC Public Health. 2011;11:239.

112. Payaprom Y, Bennett P, Alabaster E, Tantipong H. Using the Health Action Process Approach and implementation intentions to increase flu vaccine uptake in high risk Thai individuals: a controlled before-after trial. Health Psychol. 2011;30(4):492-500.

113. Piedimonte S, Leung A, Zakhari A, Giordano C, Tellier PP, Lau S. Impact of an HPV Education and Vaccination Campaign among Canadian University Students. J Obstet Gynaecol Can. 2018;40(4):440-6.

114. Powell-Jackson T, Fabbri C, Dutt V, Tougher S, Singh K. Effect and cost-effectiveness of educating mothers about childhood DPT vaccination on immunisation uptake, knowledge, and perceptions in Uttar Pradesh, India: A randomised controlled trial. PLoS Med. 2018;15(3):e1002519.

115. Reiter PL, Katz ML, Bauermeister JA, Shoben AB, Paskett ED, McRee AL. Increasing Human Papillomavirus Vaccination Among Young Gay and Bisexual Men: A Randomized Pilot Trial of the Outsmart HPV Intervention. LGBT Health. 2018;5(5):325-9.

116. Richman AR, Maddy L, Torres E, Goldberg EJ. A randomized intervention study to evaluate whether electronic messaging can increase human papillomavirus vaccine completion and knowledge among college students. J Am Coll Health. 2016;64(4):269-78.

117. Rodriguez RM, Baraff LJ. Emergency department immunization of the elderly with pneumococcal and influenza vaccines. Ann Emerg Med. 1993;22(11):1729-32.

118. Rothan-Tondeur M, Filali-Zegzouti Y, Belmin J, Lejeune B, Golmard JL, de Wazieres B, et al. Assessment of healthcare worker influenza vaccination program in French geriatric wards: a cluster-randomized controlled trial. Aging Clin Exp Res. 2010;22(5-6):450-5.

119. Saitoh A, Nagata S, Saitoh A, Tsukahara Y, Vaida F, Sonobe T, et al. Perinatal immunization education improves immunization rates and knowledge: a randomized controlled trial. Prev Med. 2013;56(6):398-405.

120. Saitoh A, Saitoh A, Sato I, Shinozaki T, Kamiya H, Nagata S. Effect of stepwise perinatal immunization education: A cluster-randomized controlled trial. Vaccine. 2017;35(12):1645-51.

121. Saitoh A, Saitoh A, Katsuta T, Mine M, Kamiya H, Miyairi I, et al. Effect of a vaccine information statement (VIS) on immunization status and parental knowledge, attitudes, and beliefs regarding infant immunization in Japan. Vaccine. 2020;38(50):8049-54.

122. Sanderson M, Canedo JR, Khabele D, Fadden MK, Harris C, Beard K, et al. Pragmatic trial of an intervention to increase human papillomavirus vaccination in safety-net clinics. BMC Public Health. 2017;17(1):158.

123. Schluter WW, Ralston DL, Delaney RJ, Sauaia A, Dunn TR. Increasing influenza and pneumococcal vaccination and tuberculosis screening among residents of Colorado long-term care facilities. Eval Health Prof. 1999;22(4):466-83.

124. Scott VP, Opel DJ, Reifler J, Rikin S, Pethe K, Barrett A, et al. Office-Based Educational Handout for Influenza Vaccination: A Randomized Controlled Trial. Pediatrics. 2019;144(2).

125. Shegog R, Savas LS, Healy CM, Frost EL, Coan SP, Gabay EK, et al. AVPCancerFree: Impact of a digital behavior change intervention on parental HPV vaccine -related perceptions and behaviors. Hum Vaccin Immunother. 2022;18(5):2087430.

126. Si M, Su X, Jiang Y, Wang W, Zhang X, Gu X, et al. An Internet-Based Education Program for Human Papillomavirus Vaccination Among Female College Students in Mainland China: Application of the Information-Motivation-Behavioral Skills Model in a Cluster Randomized Trial. J Med Internet Res. 2022;24(9):e37848.

127. Stille CJ, Christison-Lagay J, Bernstein BA, Dworkin PH. A simple provider-based educational intervention to boost infant immunization rates: a controlled trial. Clin Pediatr (Phila). 2001;40(7):365-73.

128. Tapiainen T, Bar G, Schaad UB, Heininger U. Influenza vaccination among healthcare workers in a university children's hospital. Infect Control Hosp Epidemiol. 2005;26(11):855-8.

129. Takamatsu A, Honda H, Kojima T, Murata K, Babcock HM. Promoting coronavirus disease 2019 (COVID-19) vaccination among healthcare personnel: A multifaceted intervention at a tertiary-care center in Japan. Infect Control Hosp Epidemiol. 2022;43(9):1201-6.

130. Thomas DM, Ray SM, Morton FJ, Drew JS, Offutt G, Whitney CG, et al. Patient education strategies to improve pneumococcal vaccination rates: randomized trial. J Investig Med. 2003;51(3):141-8.

131. Thomas CM, Loewen A, Coffin C, Campbell NR. Improving rates of pneumococcal vaccination on discharge from a tertiary center medical teaching unit: a prospective intervention. BMC Public Health. 2005;5:110.

132. Tisi G, Salinaro F, Apostoli P, Bassani R, Bellicini A, Groppi L, et al. HPV vaccination acceptability in young boys. Ann Ist Super Sanita. 2013;49(3):286-91.

133. Tubeuf S, Edlin R, Shourie S, Cheater FM, Bekker H, Jackson C. Cost effectiveness of a web-based decision aid for parents deciding about MMR vaccination: a three-arm cluster randomised controlled trial in primary care. Br J Gen Pract. 2014;64(625):e493-9.

134. Underwood NL, Gargano LM, Sales J, Vogt TM, Seib K, Hughes JM. Evaluation of Educational Interventions to Enhance Adolescent Specific Vaccination Coverage. J Sch Health. 2019;89(8):603-11.

135. Usami T, Hashiguchi M, Kouhara T, Ishii A, Nagata T, Mochizuki M. Impact of community pharmacists advocating immunization on influenza vaccination rates among the elderly. Yakugaku Zasshi. 2009;129(9):1063-8.

136. Usman HR, Akhtar S, Habib F, Jehan I. Redesigned immunization card and center-based education to reduce childhood immunization dropouts in urban Pakistan: a randomized controlled trial. Vaccine. 2009;27(3):467-72.

137. Usman HR, Rahbar MH, Kristensen S, Vermund SH, Kirby RS, Habib F, et al. Randomized controlled trial to improve childhood immunization adherence in rural Pakistan: redesigned immunization card and maternal education. Trop Med Int Health. 2011;16(3):334-42.

138. Van Amburgh JA, Waite NM, Hobson EH, Migden H. Improved influenza vaccination rates in a rural population as a result of a pharmacist-managed immunization campaign. Pharmacotherapy. 2001;21(9):1115-22.

139. Watson B. Hepatitis B immunization of Asian Pacific Islanders in the United States. Pediatr Infect Dis J. 1998;17(7 Suppl):S38-42.

140. Weaver FM, Smith B, LaVela S, Wallace C, Evans CT, Hammond M, et al. Interventions to increase influenza vaccination rates in veterans with spinal cord injuries and disorders. J Spinal Cord Med. 2007;30(1):10-9.

141. Webb NJ, Lindsley J, Stockbridge EL, Workman A, Reynolds CD, Miller TL, et al. Effectiveness of an intervention to overcome influenza vaccine hesitancy in specialty clinic patients. Medicine (Baltimore). 2022;101(30):e29786.

142. Wedel S, Navarrete R, Burkard JF, Clark MJ. Improving Human Papillomavirus Vaccinations in Military Women. Mil Med. 2016;181(10):1224-7.

143. Williams SE, Rothman RL, Offit PA, Schaffner W, Sullivan M, Edwards KM. A randomized trial to increase acceptance of childhood vaccines by vaccine-hesitant parents: a pilot study. Acad Pediatr. 2013;13(5):475-80.

144. Winer RL, Gonzales AA, Noonan CJ, Buchwald DS. A Cluster-Randomized Trial to Evaluate a Mother-Daughter Dyadic Educational Intervention for Increasing HPV Vaccination Coverage in American Indian Girls. J Community Health. 2016;41(2):274-81.

145. Wong VWY, Fong DYT, Lok KYW, Wong JYH, Sing C, Choi AY, et al. Brief education to promote maternal influenza vaccine uptake: A randomized controlled trial. Vaccine. 2016;34(44):5243-50.

146. Berenbrok LA, Gessler C, Kirisci L, Herrera-Restrepo O, Coley KC. Impact of pharmacist motivational interviewing on hepatitis B vaccination in adults with diabetes. J Am Pharm Assoc (2003). 2023;63(1):66-73 e1.

147. Real FJ, DeBlasio D, Beck AF, Ollberding NJ, Davis D, Cruse B, et al. A Virtual Reality Curriculum for Pediatric Residents Decreases Rates of Influenza Vaccine Refusal. Acad Pediatr. 2017;17(4):431-5.

S3. Search syntax

("behaviour*" [Title/Abstract] OR "behavior*" [Title/Abstract] OR "communication*" [Title/Abstract] OR "motivation*" [Title/Abstract] OR behaviour[Mesh] OR "education*" [Title/Abstract] OR "vaccine information" [Title/Abstract] OR "education"[Mesh]) AND ("intervention*" [Title/Abstract] OR "trial*" [Title/Abstract] OR "initiative*" [Title/Abstract] OR "effort*" [Title/Abstract] OR "project*" [Title/Abstract] OR "campaign*" [Title/Abstract] OR "program*" [Title/Abstract] OR "counseling" [Title/Abstract] OR "counselling" [Title/Abstract] OR "counseling"[Mesh] OR "interview*" [Title/Abstract] OR "Motivational Interviewing"[Mesh]) AND ("vaccin*" [Title/Abstract] OR "immunisation*" [Title/Abstract] OR "immunization*" [Title/Abstract] OR Vaccination[Mesh])

S4. Funnel plot of motivational interviewing/communication (MI/MC) intervention studies (Randomized Controlled Trials only) describing risk of publication bias


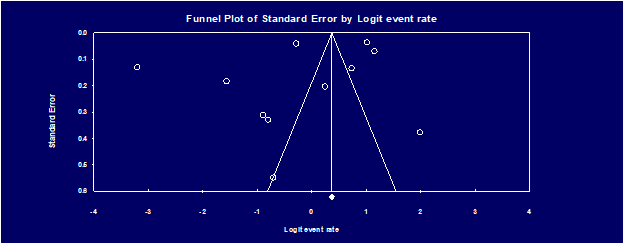


S5. Funnel plot of Educational studies (Randomized Controlled Trials only) describing risk of publication bias


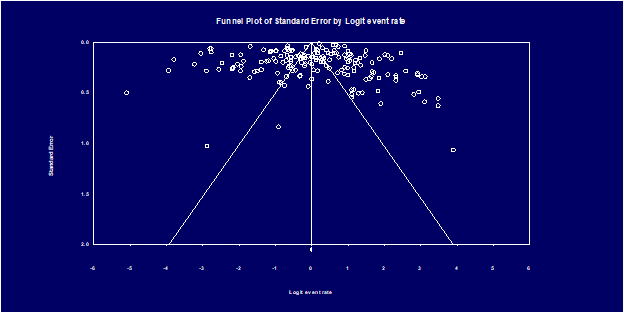


S6 Quality assessment scoring according to Down and Blake checklist


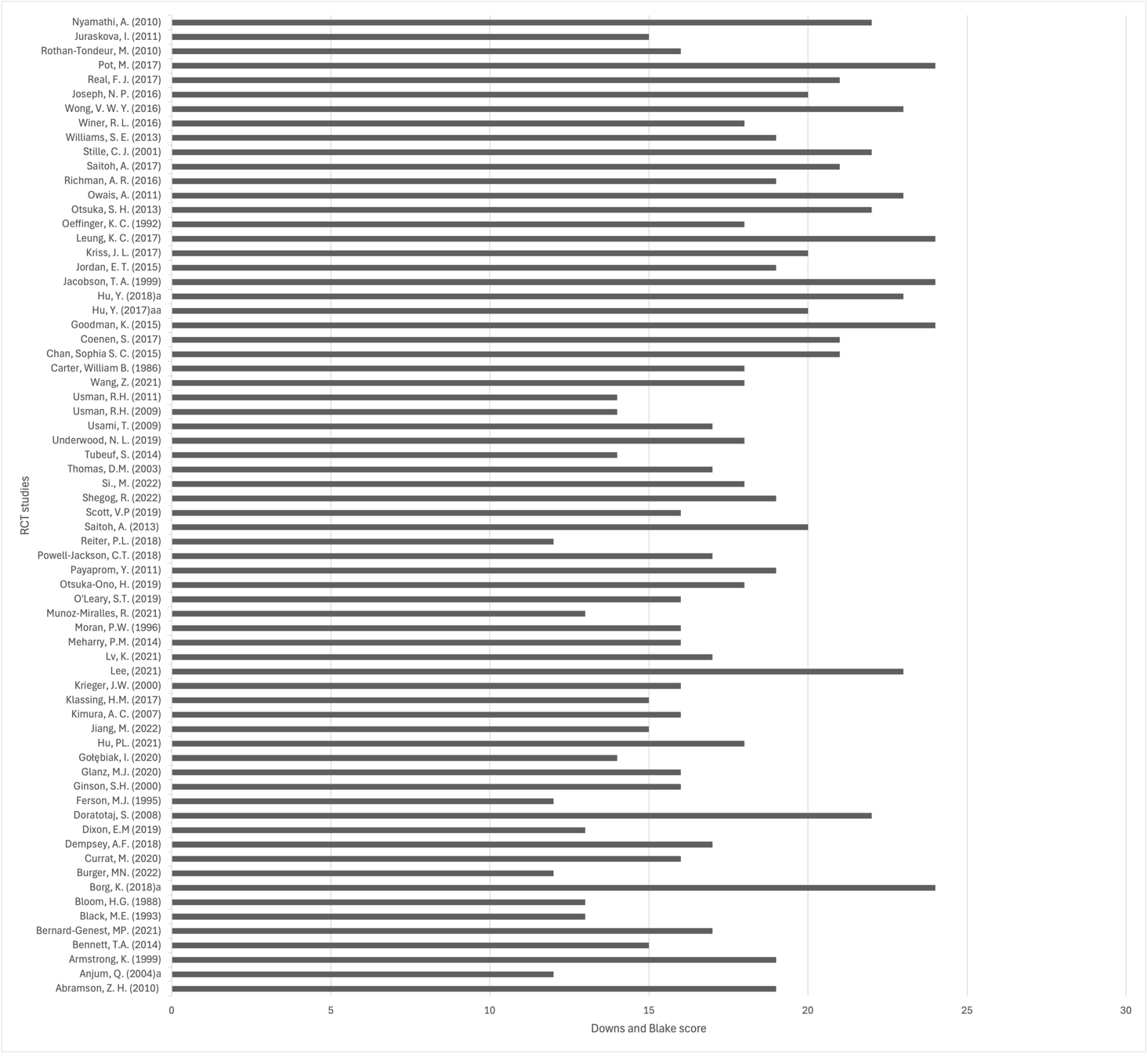


1. Abramson ZH, Avni O, Levi O, Miskin IN. Randomized trial of a program to increase staff influenza vaccination in primary care clinics. Ann Fam Med. 2010;8(4):293-8.

2. Abu-Rish EY, Barakat NA. The impact of pharmacist-led educational intervention on pneumococcal vaccine awareness and acceptance among elderly in Jordan. Hum Vaccin Immunother. 2021;17(4):1181-9.

3. Altay M, Ates I, Altay FA, Kaplan M, Akca O, Ozkara A. Does education effect the rates of prophylactic vaccination in elderly diabetics? Diabetes Res Clin Pract. 2016;120:117-23.

4. Schattner A. Cardiovascular-targeted patient education and uptake of influenza vaccination in elderly patients. Patient Educ Couns. 2020;103(5):1052-4.

5. Anjum Q, Omair A, Inam SN, Ahmed Y, Usman Y, Shaikh S. Improving vaccination status of children under five through health education. J Pak Med Assoc. 2004;54(12):610-3.

6. Aragones A, Bruno DM, Ehrenberg M, Tonda-Salcedo J, Gany FM. Parental education and text messaging reminders as effective community based tools to increase HPV vaccination rates among Mexican American children. Prev Med Rep. 2015;2:554-8.

7. Armstrong K, Berlin M, Schwartz JS, Propert K, Ubel PA. Educational content and the effectiveness of influenza vaccination reminders. J Gen Intern Med. 1999;14(11):695-8.

8. Baltes M, Pronovost E, Kanuch S, Caron A. Improving Influenza Vaccination Rates in an Underserved Primary Care Clinic Using a Simple Educational Tool. Qual Manag Health Care. 2022;31(2):80-4.

9. Bennett AT, Patel DA, Carlos RC, Zochowski MK, Pennewell SM, Chi AM, et al. Human Papillomavirus Vaccine Uptake After a Tailored, Online Educational Intervention for Female University Students: A Randomized Controlled Trial. J Womens Health (Larchmt). 2015;24(11):950-7.

10. Bernard-Genest MP, Ruel-Laliberte J, Lapointe-Milot K. Effect of educative reminder telephone calls on human papillomavirus immunization rate: A randomized controlled trial. Womens Health (Lond). 2021;17:17455065211003821.

11. Bernstein HH, Monty M, Yang P, Cohen A. Increasing Tdap Coverage Among Postpartum Women: A Quality Improvement Intervention. Pediatrics. 2017;139(3).

12. Bertin M, Scarpelli M, Proctor AW, Sharp J, Robitson E, Donnelly T, et al. Novel use of the intranet to document health care personnel participation in a mandatory influenza vaccination reporting program. Am J Infect Control. 2007;35(1):33-7.

13. Bielecki K, Craig J, Willocks LJ, Pollock KG, Gorman DR. Impact of an influenza information pamphlet on vaccination uptake among Polish pupils in Edinburgh, Scotland and the role of social media in parental decision making. BMC Public Health. 2020;20(1):1381.

14. Black ME, Ploeg J, Walter SD, Hutchinson BG, Scott EA, Chambers LW. The impact of a public health nurse intervention on influenza vaccine acceptance. Am J Public Health. 1993;83(12):1751-3.

15. Bloom HG, Bloom JS, Krasnoff L, Frank AD. Increased utilization of influenza and pneumococcal vaccines in an elderly hospitalized population. J Am Geriatr Soc. 1988;36(10):897-901.

16. Borg K, Sutton K, Beasley M, Tull F, Faulkner N, Halliday J, et al. Communication-based interventions for increasing influenza vaccination rates among Aboriginal children: A randomised controlled trial. Vaccine. 2018;36(45):6790-5.

17. Bryant KA, Stover B, Cain L, Levine GL, Siegel J, Jarvis WR. Improving influenza immunization rates among healthcare workers caring for high-risk pediatric patients. Infect Control Hosp Epidemiol. 2004;25(11):912-7.

18. Burger MN, Mayer M, Steimanis I. Repeated information of benefits reduces COVID-19 vaccination hesitancy: Experimental evidence from Germany. PloS one. 2022;17(6):e0270666.

19. Bushar JA, Kendrick JS, Ding H, Black CL, Greby SM. Text4baby Influenza Messaging and Influenza Vaccination Among Pregnant Women. Am J Prev Med. 2017;53(6):845-53.

20. Carter WB, Beach LR, Inui TS. The flu shot study: using multiattribute utility theory to design a vaccination intervention. Organ Behav Hum Decis Process. 1986;38(3):378-91.

21. Cassidy B, Braxter B, Charron-Prochownik D, Schlenk EA. A quality improvement initiative to increase HPV vaccine rates using an educational and reminder strategy with parents of preteen girls. J Pediatr Health Care. 2014;28(2):155-64.

22. Cassidy WM, Mahoney FJ. A hepatitis B vaccination program targeting adolescents. J Adolesc Health. 1995;17(4):244-7.

23. Cebollero J, Walton SM, Cavendish L, Quairoli K, Cwiak C, Kottke MJ. Evaluation of Human Papillomavirus Vaccination After Pharmacist-Led Intervention: A Pilot Project in an Ambulatory Clinic at a Large Academic Urban Medical Center. Public Health Rep. 2020;135(3):313-21.

24. Chan SS, Leung DY, Leung AY, Lam C, Hung I, Chu D, et al. A nurse-delivered brief health education intervention to improve pneumococcal vaccination rate among older patients with chronic diseases: a cluster randomized controlled trial. Int J Nurs Stud. 2015;52(1):317-24.

25. Cheng PJ, Huang SY, Su SY, Peng HH, Chang CL. Increasing postpartum rate of vaccination with tetanus, diphtheria, and acellular pertussis vaccine by incorporating pertussis cocooning information into prenatal education for group B streptococcus prevention. Vaccine. 2015;33(51):7225-31.

26. Coenen S, Weyts E, Jorissen C, De Munter P, Noman M, Ballet V, et al. Effects of Education and Information on Vaccination Behavior in Patients with Inflammatory Bowel Disease. Inflamm Bowel Dis. 2017;23(2):318-24.

27. Colon-Lopez V, Ayala-Marin A, Velez-Alamo C, Soto-Salgado M, Medina-Cortes L, Acevedo-Fontanez AI, et al. inverted exclamation markHabla de VPH! An Educational Activity for College Students in Puerto Rico. P R Health Sci J. 2021;40(3):142-6.

28. Collins L, Dupont L, Nagle D. The impact of educational efforts on first-year university students' acceptance of meningococcal vaccine. J Am Coll Health. 2003;52(1):41-3.

29. Costantino C, Restivo V, Gaglio V, Lanza GLM, Marotta C, Maida CM, et al. Effectiveness of an educational intervention on seasonal influenza vaccination campaign adherence among healthcare workers of the Palermo University Hospital, Italy. Ann Ig. 2019;31(1):35-44.

30. Costantino C, Mazzucco W, Bonaccorso N, Cimino L, Conforto A, Sciortino M, et al. Educational Interventions on Pregnancy Vaccinations during Childbirth Classes Improves Vaccine Coverages among Pregnant Women in Palermo's Province. Vaccines (Basel). 2021;9(12).

31. Currat M, Lazor-Blanchet C, Zanetti G. Promotion of the influenza vaccination to hospital staff during pre-employment health check: a prospective, randomised, controlled trial. J Occup Med Toxicol. 2020;15(1):34.

32. Deuson RR, Brodovicz KG, Barker L, Zhou F, Euler GL. Economic analysis of a child vaccination project among Asian Americans in Philadelphia, Pa. Arch Pediatr Adolesc Med. 2001;155(8):909-14.

33. Dixon BE, Zimet GD, Xiao S, Tu W, Lindsay B, Church A, et al. An Educational Intervention to Improve HPV Vaccination: A Cluster Randomized Trial. Pediatrics. 2019;143(1).

34. Doratotaj S, Macknin ML, Worley S. A novel approach to improve influenza vaccination rates among health care professionals: a prospective randomized controlled trial. Am J Infect Control. 2008;36(4):301-3.

35. Drokow EK, Effah CY, Agboyibor C, Sasu E, Amponsem-Boateng C, Akpabla GS, et al. The Impact of Video-Based Educational Interventions on Cervical Cancer, Pap Smear and HPV Vaccines. Front Public Health. 2021;9:681319.

36. Elangovan S, Kallail KJ, Vargo G. Improving pneumococcal vaccination rates in an elderly population by patient education in an outpatient clinic. J Am Board Fam Pract. 1996;9(6):411-3.

37. Ferson MJ, Fitzsimmons G, Christie D, Woollett H. School health nurse interventions to increase immunisation uptake in school entrants. Public Health. 1995;109(1):25-9.

38. Fuchs J. The provision of pharmaceutical advice improves patient vaccination status. Pharm Pract (Granada). 2006;4(4):163-7.

39. Ginson HS, Malmberg C, French JD. Impact on Vaccination Rates of a Pharmacist-Initiated Influenza and Pneumococcal Vaccination Program. The Canadian Journal of Hospital Pharmacy. 2000;53.

40. Glanz JM, Wagner NM, Narwaney KJ, Pyrzanowski J, Kwan BM, Sevick C, et al. Web-Based Tailored Messaging to Increase Vaccination: A Randomized Clinical Trial. Pediatrics. 2020;146(5).

41. Golebiak I, Okreglicka K, Kanecki K, Nitsch-Osuch A. The impact of selected educational and information interventions on the coverage rate and attitudes to influenza vaccination in nursing staff. Med Pr. 2020;71(6):665-85.

42. Goodman K, Mossad SB, Taksler GB, Emery J, Schramm S, Rothberg MB. Impact of Video Education on Influenza Vaccination in Pregnancy. J Reprod Med. 2015;60(11-12):471-9.

43. Hohmeier KC, Randolph DD, Smith CT, Hagemann TM. A multimodal approach to improving human papillomavirus vaccination in a community pharmacy setting. SAGE Open Med. 2016;4:2050312116682128.

44. Hu Y, Chen Y, Wang Y, Song Q, Li Q. Prenatal vaccination education intervention improves both the mothers' knowledge and children's vaccination coverage: Evidence from randomized controlled trial from eastern China. Hum Vaccin Immunother. 2017;13(6):1-8.

45. Hu Y, Li Q, Chen Y. Evaluation of two health education interventions to improve the varicella vaccination: a randomized controlled trial from a province in the east China. BMC Public Health. 2018;18(1):144.

46. Ho HJ, Tan YR, Cook AR, Koh G, Tham TY, Anwar E, et al. Increasing Influenza and Pneumococcal Vaccination Uptake in Seniors Using Point-of-Care Informational Interventions in Primary Care in Singapore: A Pragmatic, Cluster-Randomized Crossover Trial. Am J Public Health. 2019;109(12):1776-83.

47. Hu PL, Koh EYL, Tay JSH, Chan VX, Goh SSM, Wang SZ. Assessing the impact of educational methods on influenza vaccine uptake and patient knowledge and attitudes: a randomised controlled trial. Singapore Med J. 2023;64(2):98-104.

48. Jacobson TA, Thomas DM, Morton FJ, Offutt G, Shevlin J, Ray S. Use of a low-literacy patient education tool to enhance pneumococcal vaccination rates. A randomized controlled trial. JAMA. 1999;282(7):646-50.

49. Jiang M, Yao X, Li P, Fang Y, Feng L, Hayat K, et al. Impact of video-led educational intervention on uptake of influenza vaccine among the elderly in western China: a community-based randomized controlled trial. BMC Public Health. 2022;22(1):1128.

50. Jimenez-Quinones EM, Melin K, Jimenez-Ramirez FJ. Impact of a Pharmacist Conducted Educational Program on Human Papilloma Virus Vaccination Rates in a Low Socioeconomic Population in the City of Lares, PR. P R Health Sci J. 2017;36(2):67-70.

51. Johnson EA, Harwell TS, Donahue PM, Weisner MA, McInerney MJ, Holzman GS, et al. Promoting pneumococcal immunizations among rural Medicare beneficiaries using multiple strategies. J Rural Health. 2003;19(4):506-10.

52. Jordan ET, Bushar JA, Kendrick JS, Johnson P, Wang J. Encouraging Influenza Vaccination Among Text4baby Pregnant Women and Mothers. Am J Prev Med. 2015;49(4):563-72.

53. Juraskova I, Bari RA, O'Brien MT, McCaffery KJ. HPV vaccine promotion: does referring to both cervical cancer and genital warts affect intended and actual vaccination behavior? Womens Health Issues. 2011;21(1):71-9.

54. Kimura AC, Nguyen CN, Higa JI, Hurwitz EL, Vugia DJ. The effectiveness of vaccine day and educational interventions on influenza vaccine coverage among health care workers at long-term care facilities. Am J Public Health. 2007;97(4):684-90.

55. Klassing HM, Ruisinger JF, Prohaska ES, Melton BL. Evaluation of Pharmacist-Initiated Interventions on Vaccination Rates in Patients with Asthma or COPD. J Community Health. 2018;43(2):297-303.

56. Krieger JW, Castorina JS, Walls ML, Weaver MR, Ciske S. Increasing influenza and pneumococcal immunization rates: a randomized controlled study of a senior center-based intervention. Am J Prev Med. 2000;18(2):123-31.

57. Kriss JL, Frew PM, Cortes M, Malik FA, Chamberlain AT, Seib K, et al. Evaluation of two vaccine education interventions to improve pertussis vaccination among pregnant African American women: A randomized controlled trial. Vaccine. 2017;35(11):1551-8.

58. Kuntz JL, Holley S, Helms CM, Cavanaugh JE, Vande Berg J, Herwaldt LA, et al. Use of a pandemic preparedness drill to increase rates of influenza vaccination among healthcare workers. Infect Control Hosp Epidemiol. 2008;29(2):111-5.

59. Lee HY, Koopmeiners JS, McHugh J, Raveis VH, Ahluwalia JS. mHealth Pilot Study: Text Messaging Intervention to Promote HPV Vaccination. Am J Health Behav. 2016;40(1):67-76.

60. Lee JL, Foschini L, Kumar S, Juusola J, Liska J, Mercer M, et al. Digital intervention increases influenza vaccination rates for people with diabetes in a decentralized randomized trial. NPJ Digit Med. 2021;4(1):138.

61. Leung KC, Mui C, Chiu WY, Ng YY, Chen MHY, Ho PH, et al. Impact of patient education on influenza vaccine uptake among community-dwelling elderly: a randomized controlled trial. Health Educ Res. 2017;32(5):455-64.

62. Lubis TA, Gunardi H, Herqutanto, Soedjatmiko S, Satari HI, Alatas FS, et al. Educational videos to address vaccine hesitancy in childhood immunization. Vaccine. 2022;40(41):5965-70.

63. Lv K, Zhao J, Zhang P. The effect of community comprehensive nursing using scenario-based health education on the infant and young child immunization rates. Am J Transl Res. 2021;13(8):9663-70.

64. Meharry PM, Cusson RM, Stiller R, Vazquez M. Maternal influenza vaccination: evaluation of a patient-centered pamphlet designed to increase uptake in pregnancy. Matern Child Health J. 2014;18(5):1205-14.

65. Moran WP, Nelson K, Wofford JL, Velez R, Case LD. Increasing influenza immunization among high-risk patients: education or financial incentive? Am J Med. 1996;101(6):612-20.

66. Munoz-Miralles R, Bonvehi Nadeu S, Sant Masoliver C, Martin Gallego A, Gomez Del Canto J, Mendioroz Pena J, et al. Effectiveness of a brief intervention for acceptance of influenza vaccine in reluctant primary care patients. Gac Sanit. 2022;36(5):446-51.

67. Nyamathi A, Sinha K, Greengold B, Cohen A, Marfisee M. Predictors of HAV/HBV vaccination completion among methadone maintenance clients. Res Nurs Health. 2010;33(2):120-32.

68. Oeffinger KC, Roaten SP, Hitchcock MA, Oeffinger PK. The effect of patient education on pediatric immunization rates. J Fam Pract. 1992;35(3):288-93.

69. O'Leary ST, Narwaney KJ, Wagner NM, Kraus CR, Omer SB, Glanz JM. Efficacy of a Web-Based Intervention to Increase Uptake of Maternal Vaccines: An RCT. Am J Prev Med. 2019;57(4):e125-e33.

70. Orgul G, Keles AI, Yucel A, Sahin D. The Rate of Influenza Vaccination after Face-to-Face Interview in Pregnancy. Z Geburtshilfe Neonatol. 2021;225(5):423-7.

71. Otsuka SH, Tayal NH, Porter K, Embi PJ, Beatty SJ. Improving herpes zoster vaccination rates through use of a clinical pharmacist and a personal health record. Am J Med. 2013;126(9):832 e1-6.

72. Otsuka-Ono H, Hori N, Ohta H, Uemura Y, Kamibeppu K. A childhood immunization education program for parents delivered during late pregnancy and one-month postpartum: a randomized controlled trial. BMC Health Serv Res. 2019;19(1):798.

73. Owais A, Hanif B, Siddiqui AR, Agha A, Zaidi AK. Does improving maternal knowledge of vaccines impact infant immunization rates? A community-based randomized-controlled trial in Karachi, Pakistan. BMC Public Health. 2011;11:239.

74. Payaprom Y, Bennett P, Alabaster E, Tantipong H. Using the Health Action Process Approach and implementation intentions to increase flu vaccine uptake in high risk Thai individuals: a controlled before-after trial. Health Psychol. 2011;30(4):492-500.

75. Piedimonte S, Leung A, Zakhari A, Giordano C, Tellier PP, Lau S. Impact of an HPV Education and Vaccination Campaign among Canadian University Students. J Obstet Gynaecol Can. 2018;40(4):440-6.

76. Powell-Jackson T, Fabbri C, Dutt V, Tougher S, Singh K. Effect and cost-effectiveness of educating mothers about childhood DPT vaccination on immunisation uptake, knowledge, and perceptions in Uttar Pradesh, India: A randomised controlled trial. PLoS Med. 2018;15(3):e1002519.

77. Reiter PL, Katz ML, Bauermeister JA, Shoben AB, Paskett ED, McRee AL. Increasing Human Papillomavirus Vaccination Among Young Gay and Bisexual Men: A Randomized Pilot Trial of the Outsmart HPV Intervention. LGBT Health. 2018;5(5):325-9.

78. Richman AR, Maddy L, Torres E, Goldberg EJ. A randomized intervention study to evaluate whether electronic messaging can increase human papillomavirus vaccine completion and knowledge among college students. J Am Coll Health. 2016;64(4):269-78.

79. Rodriguez RM, Baraff LJ. Emergency department immunization of the elderly with pneumococcal and influenza vaccines. Ann Emerg Med. 1993;22(11):1729-32.

80. Rothan-Tondeur M, Filali-Zegzouti Y, Belmin J, Lejeune B, Golmard JL, de Wazieres B, et al. Assessment of healthcare worker influenza vaccination program in French geriatric wards: a cluster-randomized controlled trial. Aging Clin Exp Res. 2010;22(5-6):450-5.

81. Saitoh A, Nagata S, Saitoh A, Tsukahara Y, Vaida F, Sonobe T, et al. Perinatal immunization education improves immunization rates and knowledge: a randomized controlled trial. Prev Med. 2013;56(6):398-405.

82. Saitoh A, Saitoh A, Sato I, Shinozaki T, Kamiya H, Nagata S. Effect of stepwise perinatal immunization education: A cluster-randomized controlled trial. Vaccine. 2017;35(12):1645-51.

83. Saitoh A, Saitoh A, Katsuta T, Mine M, Kamiya H, Miyairi I, et al. Effect of a vaccine information statement (VIS) on immunization status and parental knowledge, attitudes, and beliefs regarding infant immunization in Japan. Vaccine. 2020;38(50):8049-54.

84. Sanderson M, Canedo JR, Khabele D, Fadden MK, Harris C, Beard K, et al. Pragmatic trial of an intervention to increase human papillomavirus vaccination in safety-net clinics. BMC Public Health. 2017;17(1):158.

85. Schluter WW, Ralston DL, Delaney RJ, Sauaia A, Dunn TR. Increasing influenza and pneumococcal vaccination and tuberculosis screening among residents of Colorado long-term care facilities. Eval Health Prof. 1999;22(4):466-83.

86. Scott VP, Opel DJ, Reifler J, Rikin S, Pethe K, Barrett A, et al. Office-Based Educational Handout for Influenza Vaccination: A Randomized Controlled Trial. Pediatrics. 2019;144(2).

87. Shegog R, Savas LS, Healy CM, Frost EL, Coan SP, Gabay EK, et al. AVPCancerFree: Impact of a digital behavior change intervention on parental HPV vaccine -related perceptions and behaviors. Hum Vaccin Immunother. 2022;18(5):2087430.

88. Si M, Su X, Jiang Y, Wang W, Zhang X, Gu X, et al. An Internet-Based Education Program for Human Papillomavirus Vaccination Among Female College Students in Mainland China: Application of the Information-Motivation-Behavioral Skills Model in a Cluster Randomized Trial. J Med Internet Res. 2022;24(9):e37848.

89. Stille CJ, Christison-Lagay J, Bernstein BA, Dworkin PH. A simple provider-based educational intervention to boost infant immunization rates: a controlled trial. Clin Pediatr (Phila). 2001;40(7):365-73.

90. Tapiainen T, Bar G, Schaad UB, Heininger U. Influenza vaccination among healthcare workers in a university children's hospital. Infect Control Hosp Epidemiol. 2005;26(11):855-8.

91. Takamatsu A, Honda H, Kojima T, Murata K, Babcock HM. Promoting coronavirus disease 2019 (COVID-19) vaccination among healthcare personnel: A multifaceted intervention at a tertiary-care center in Japan. Infect Control Hosp Epidemiol. 2022;43(9):1201-6.

92. Thomas DM, Ray SM, Morton FJ, Drew JS, Offutt G, Whitney CG, et al. Patient education strategies to improve pneumococcal vaccination rates: randomized trial. J Investig Med. 2003;51(3):141-8.

93. Thomas CM, Loewen A, Coffin C, Campbell NR. Improving rates of pneumococcal vaccination on discharge from a tertiary center medical teaching unit: a prospective intervention. BMC Public Health. 2005;5:110.

94. Tisi G, Salinaro F, Apostoli P, Bassani R, Bellicini A, Groppi L, et al. HPV vaccination acceptability in young boys. Ann Ist Super Sanita. 2013;49(3):286-91.

95. Tubeuf S, Edlin R, Shourie S, Cheater FM, Bekker H, Jackson C. Cost effectiveness of a web-based decision aid for parents deciding about MMR vaccination: a three-arm cluster randomised controlled trial in primary care. Br J Gen Pract. 2014;64(625):e493-9.

96. Underwood NL, Gargano LM, Sales J, Vogt TM, Seib K, Hughes JM. Evaluation of Educational Interventions to Enhance Adolescent Specific Vaccination Coverage. J Sch Health. 2019;89(8):603-11.

97. Usami T, Hashiguchi M, Kouhara T, Ishii A, Nagata T, Mochizuki M. Impact of community pharmacists advocating immunization on influenza vaccination rates among the elderly. Yakugaku Zasshi. 2009;129(9):1063-8.

98. Usman HR, Akhtar S, Habib F, Jehan I. Redesigned immunization card and center-based education to reduce childhood immunization dropouts in urban Pakistan: a randomized controlled trial. Vaccine. 2009;27(3):467-72.

99. Usman HR, Rahbar MH, Kristensen S, Vermund SH, Kirby RS, Habib F, et al. Randomized controlled trial to improve childhood immunization adherence in rural Pakistan: redesigned immunization card and maternal education. Trop Med Int Health. 2011;16(3):334-42.

100. Van Amburgh JA, Waite NM, Hobson EH, Migden H. Improved influenza vaccination rates in a rural population as a result of a pharmacist-managed immunization campaign. Pharmacotherapy. 2001;21(9):1115-22.

101. Wang Z, Lau JTF, Ip TKM, Yu Y, Fong F, Fang Y, et al. Two Web-Based and Theory-Based Interventions With and Without Brief Motivational Interviewing in the Promotion of Human Papillomavirus Vaccination Among Chinese Men Who Have Sex With Men: Randomized Controlled Trial. J Med Internet Res. 2021;23(2):e21465.

102. Watson B. Hepatitis B immunization of Asian Pacific Islanders in the United States. Pediatr Infect Dis J. 1998;17(7 Suppl):S38-42.

103. Weaver FM, Smith B, LaVela S, Wallace C, Evans CT, Hammond M, et al. Interventions to increase influenza vaccination rates in veterans with spinal cord injuries and disorders. J Spinal Cord Med. 2007;30(1):10-9.

104. Webb NJ, Lindsley J, Stockbridge EL, Workman A, Reynolds CD, Miller TL, et al. Effectiveness of an intervention to overcome influenza vaccine hesitancy in specialty clinic patients. Medicine (Baltimore). 2022;101(30):e29786.

105. Wedel S, Navarrete R, Burkard JF, Clark MJ. Improving Human Papillomavirus Vaccinations in Military Women. Mil Med. 2016;181(10):1224-7.

106. Williams SE, Rothman RL, Offit PA, Schaffner W, Sullivan M, Edwards KM. A randomized trial to increase acceptance of childhood vaccines by vaccine-hesitant parents: a pilot study. Acad Pediatr. 2013;13(5):475-80.

107. Winer RL, Gonzales AA, Noonan CJ, Buchwald DS. A Cluster-Randomized Trial to Evaluate a Mother-Daughter Dyadic Educational Intervention for Increasing HPV Vaccination Coverage in American Indian Girls. J Community Health. 2016;41(2):274-81.

108. Wong VWY, Fong DYT, Lok KYW, Wong JYH, Sing C, Choi AY, et al. Brief education to promote maternal influenza vaccine uptake: A randomized controlled trial. Vaccine. 2016;34(44):5243-50.

109. Berenbrok LA, Gessler C, Kirisci L, Herrera-Restrepo O, Coley KC. Impact of pharmacist motivational interviewing on hepatitis B vaccination in adults with diabetes. J Am Pharm Assoc (2003). 2023;63(1):66-73 e1.

110. Jamison KC, Ahmed AH, Spoerner DA, Kinney D. Best shot: A motivational interviewing approach to address vaccine hesitancy in pediatric outpatient settings. J Pediatr Nurs. 2022;67:124-31.

111. Joseph NP, Bernstein J, Pelton S, Belizaire M, Goff G, Horanieh N, et al. Brief Client-Centered Motivational and Behavioral Intervention to Promote HPV Vaccination in a Hard-to-Reach Population: A Pilot Randomized Controlled Trial. Clin Pediatr (Phila). 2016;55(9):851-9.

112. Labbe S, Colmegna I, Valerio V, Boucher VG, Pelaez S, Dragomir AI, et al. Training Physicians in Motivational Communication to Address Influenza Vaccine Hesitation: A Proof-of-Concept Study. Vaccines (Basel). 2022;10(2).

113. Real FJ, DeBlasio D, Beck AF, Ollberding NJ, Davis D, Cruse B, et al. A Virtual Reality Curriculum for Pediatric Residents Decreases Rates of Influenza Vaccine Refusal. Acad Pediatr. 2017;17(4):431-5.

114. Gagneur A, Lemaitre T, Gosselin V, Farrands A, Carrier N, Petit G, et al. A postpartum vaccination promotion intervention using motivational interviewing techniques improves short-term vaccine coverage: PromoVac study. BMC Public Health. 2018;18(1):811.

115. Dempsey AF, Pyrznawoski J, Lockhart S, Barnard J, Campagna EJ, Garrett K, et al. Effect of a Health Care Professional Communication Training Intervention on Adolescent Human Papillomavirus Vaccination: A Cluster Randomized Clinical Trial. JAMA Pediatr. 2018;172(5):e180016.

116. Pot M, Paulussen TG, Ruiter RA, Eekhout I, de Melker HE, Spoelstra ME, et al. Effectiveness of a Web-Based Tailored Intervention With Virtual Assistants Promoting the Acceptability of HPV Vaccination Among Mothers of Invited Girls: Randomized Controlled Trial. J Med Internet Res. 2017;19(9):e312.
